# The Role of Autophagy in Tumor Immune Infiltration in Colorectal Cancer

**DOI:** 10.1155/2022/2055676

**Published:** 2022-03-14

**Authors:** Yu Bian-fang, Wu Dong-ning, Teng Dan, Shi Jian-yu, Wang Shi-yi, Wang Ben-jun, Dong Xin, Zhao Wen-wen, Wang Qing-feng, Zhao Yan

**Affiliations:** ^1^Department of Proctology, Affiliated Hospital of Shandong University of Traditional Chinese Medicine, Jinan, Shandong, China; ^2^Clinical Evaluation Center, Chinese Academy of Chinese Medical Sciences, Beijing, China; ^3^Artificial Intelligence and Big Data College, HE University, Shenyang, Liaoning, China; ^4^Department of Proctology, Ping Yi People's Hospital, Linyi, Shandong, China; ^5^Liaoning University of Traditional Chinese Medicine, Shenyang, Liaoning, China; ^6^Department of Rehabilitation, Dong Ying People's Hospital, Dongying, Shandong, China; ^7^The Second Affiliated Hospital of Liaoning University of Traditional Chinese Medicine, Shenyang, Liaoning, China

## Abstract

**Objective:**

This study is aimed at exploring the association between autophagy and tumor immune infiltration (TII) in colorectal cancer (CRC).

**Methods and Materials:**

We downloaded the transcriptome profiling and clinical data for CRC from The Cancer Genome Atlas (TCGA) database and obtained the normal colon transcriptome profiling data from Genotype-Tissue Expression Project (GTEx) database. The list of autophagy-related signatures was obtained from the Human Autophagy Database. We isolated the autophagy-related genes from the CRC gene expression matrix and constructed an autophagy-related prognostic (ARP) risk model. Then, we constructed a multiROC curve to validate the prognostic ability of the ARP risk model. CIBERSORT was used to determine the fractions of 22 immune cells in each CRC sample, and the association between these TII cells and CRC clinical variables was further investigated. Finally, we estimated the association of 3 hub-ARP signatures and 20 different types of TII cell distribution.

**Results:**

We classified 447 CRC patients into 224 low-risk and 223 high-risk patients using the median ARP risk score. According to the univariate survival test results, except for gender (*P* = 0.672), age (*P* = 0.008), cancer stage, and pathological stage T, M, and N were closely correlated with the prognosis of CRC patients (*P* < 0.001). Multivariate survival analysis results indicate that age and rescore were the only independent prognostic indicators with significant differences (*P* < 0.05). After merging the immune cell distribution (by CIBERSORT) with the CRC clinical data, the results indicate that activated macrophage M0 cells exhibited the highest clinical response, which included cancer stage and stage T, N, and M. Additionally, six immune cells were closely associated with cancer stage, including regulatory T cells (Tregs), gamma delta T cells, follicular helper T cells, activated memory CD4 T cells, activated NK cells, and resting dendritic cells. Finally, we evaluated the correlation of ARP signatures with TII cell distribution. Compared with the other correlation, NRG1 and plasma cells (↑), risk score and macrophage M1 (↑), NRG1 and dendritic cell activated (↑), CDKN2A and T cell CD4 memory resting (↓), risk score and T cell CD8 (↑), risk score and T cell CD4 memory resting (↓), and DAPK1 and T cell CD4 memory activated (↓) exhibited a stronger association (*P* < 0.0001).

**Conclusions:**

In summary, we explored the correlation between the risk of autophagy and the TII microenvironment in CRC patients. Furthermore, we integrated different CAR signatures with tumor-infiltrating immune cells and found robust associations between different levels of CAR signature expression and immune cell infiltrating density.

## 1. Introduction

According to global cancer statistics from 2018 [1], colorectal cancer (CRC) represents the third most commonly diagnosed cancer (6.1% of the total cases) and the second highest cause of cancer-associated death (9.2% of the total cancer deaths) throughout the world. Due to its highly aggressive nature, the rate of distant metastasis in advanced CRC is as high as 70%-80% [2], which is responsible for the high mortality among CRC patients [3]. Therefore, a better understanding of the progression, invasion, and metastasis of CRC is important for its clinical diagnosis, prognosis, and molecular targeted therapy. However, although the pathological mechanism of CRC is well studied, the complex regulatory mechanisms of CRC [4] limit the ability of research into a single molecule or a single pathway to reveal the bioregulatory landscape of CRC. Accordingly, an approach using multiple different mechanisms/phenotype coanalysis of CRC through the crosstalk of different phenotypes may provide a more reliable and accurate regulatory biomarker, which can be considered a hub of therapeutic signatures. Moreover, this hub of therapeutic signatures can potentially be utilized as predictive and therapeutic targets for CRC treatment [5, 6].

The genomic results of CRC indicate that [7, 8] compared with adjacent normal samples, as excessive tumor cell proliferation is associated with autophagy, which is involved in modification of the tumor immune microenvironment, CRC tissues exhibit greater activation of abnormal autophagy and immune infiltration. Thus, a systemic research focus on the biofunction of autophagy-related signatures in tumor immune infiltration (TII) could provide novel insight and targets for precision treatment of CRC, especially the identification of potential novel immunotherapy targets in CRC.

According to previous reports, the inhibition of autophagy in TNBC cell lines induces the secretion of the macrophage migration inhibitory factor (MIF), thereby promoting breast cancer invasion and immunomodulation [9]. Although the autophagy of B cells remains poorly understood in tumor pathogenesis, B cell activation is induced by tumor-derived autophagosomes (Dribbles), which sequester various tumor antigens in a TLR4/MYD88-dependent manner [10]. Accordingly, autophagy-TII research has introduced a new pathway that illustrates the association between autophagy and the TII microenvironment. Therefore, it is essential to confirm the relationship of specific biomarkers of autophagy and CRC. Based on the landscape of the CRC immune microenvironment, the correlation between these autophagy-related hub DEGs and TII cells is also required for research into the molecular mechanism by which autophagy-TII provides a theoretical basis for CRC clinical diagnosis and treatment. It is also possible to further regulate the downstream immune response upstream of CRC onset through autophagy-related targets, thus improving the level of immune tolerance and escape caused by disordered autophagy. This sheds new light into CRC immunotherapy and further improves the clinical treatment effect in CRC patients.

In the present study, we screened the autophagy-related risk signatures for CRC based on a bioinformatics analysis from The Cancer Genome Atlas (TCGA) and Genotype-Tissue Expression Project (GTEx) database and then evaluated the clinical value of these signatures based on multivariate Cox regression results. Next, we established an autophagy-related prognostic (ARP) risk model and conducted a correlation analysis between this risk model and its predictive efficacy in CRC. To further investigate the potential mechanisms between autophagy and TII based on TCGA, we obtained 22 types of TII cell profiles using the CIBERSORT tool. We then conducted a series of correlation analyses and evaluated the association between these TII cells and the clinical characteristics of CRC. Finally, we analyzed the correlation between proportion of TII cells and hub-ARP signatures. Therefore, we revealed the relationship between autophagy and TII regulatory characteristics of CRC and the effect of different ARP signatures in the tumor microenvironment.

## 2. Materials and Methods

### 2.1. Data Acquisition and Processing

The CRC genome expression data was downloaded from TCGA database (https://portal.gdc.cancer.gov/), which included 473 tumor samples and 41 matched normal samples. To increase the sample size, we enrolled another 384 normal colon tissue profiles from the GTEx database (https://www.gtexportal.org/). We merged the gene expression and clinical profiles of these 898 CRC patients by perl, thereby establishing the genomic and clinical database for further research. A list of autophagy-related signatures was obtained from the Human Autophagy Database (http://www.autophagy.lu/clustering/index.html), which provided an available resource for autophagy research.

### 2.2. Exploration of Autophagy in CRC Patients

First, we normalized the CRC gene expression data using Limma package by screening the abnormally expressed genes in the tumor versus normal samples. Then, we obtained the autophagy-related genes from CRC gene expression matrix by perl. The ggplot2, ggrepel, ggthemes, gridExtra, pheatmap, and ggpubr packages were used to conduct a series differential analyses (illustrated by volcano, heat map, and box figures), in which the expression differences were characterized by FDR < 0.05 and ∣logFC  | >1. Finally, we transferred the gene symbol with entrez ID and conducted Gene Ontology (GO) and KEGG analyses using the clusterProfiler, org.Hs.eg.db, enrichplot, and ggplot2 packages with *P* ≤ 0.05.

Then, we merged the futime, fustat, and gene expression matrix using perl and performed a univariate Cox regression analysis (by survival package). With the selected significant signatures (*P* ≤ 0.05), we conducted a multivariate Cox analysis and constructed the ARP risk model (using survival package). The ARP risk model was calculated as *∑*(*β*_*i*_∗Exp_*i*_), where *β*_*i*_ represented the weight of the respective signatures and Exp_*i*_ represented the expression value. Accordingly, we calculated the risk score of each patient using the median value as the cutoff value and classified patients into either a high- or low-risk group. Using the reshape2, ggplot2, scales, and cowplot packages, we established the CRC patients' vital status distribution and the expression of autophagy-related signatures in the two risk groups.

In order to assess the associations of the ARP risk model with clinical variables, we conducted the univariate and multivariate Cox regression analyses with survival and forestplot packages, with a Wilcoxon rank-sum test (two groups' comparison) or Kruskal-Wallis test (when dealing with three or more groups) results by survival and forestplot packages; we investigate the underlying relationships between the ARP risk score and clinical features (i.e., age, gender, pathological stage, and TNM stage). Then, to validate the prognostic ability of the ARP risk model, we constructed a multiROC curve to illustrate the OS prediction via survivalROC package, and AUC value was utilized to determine the predictive effect of the ARP risk score on the clinical features. A Kaplan-Meier analysis with a log-rank test was used to assess the survival differences between the two risk groups. Finally, beeswarm package was used to validate the prognostic ability of the hub-ARP signatures in relation to the clinical variables. We classified the clinical variables into two groups (age: ≤65 vs. >65, gender: female vs. male, stage: stage I-II vs. stage III-IV, T: T1-2 vs. T3-4, M: M0 vs. M1, and N: N0 vs. N1-2) and conducted a correlation analysis. We presented the *P* value (*P* value < 0.05 was considered to be statistically significant) of hub-ARP signature expression versus CRC clinical variables with beeswarm plots.

### 2.3. Exploration of TII in CRC Patients

Given the importance of TII in the pathogenesis and progression of the CRC microenvironment, we utilized CIBERSORT to determine the fractions of 22 immune cells in each sample. The TII cell constitution was presented as a bar plot. Furthermore, we conducted a Wilcoxon rank-sum test to compare the differential abundance of immune cells in the tumor and normal samples via pheatmap package, where the colors ranging from green to dark red represented low to high infiltrating levels, respectively. Finally, we illustrated the TII cell distribution in the tumor and normal samples by a violin plot.

Since we obtained the TII cell distribution in the CRC samples, we next sought to further investigate the association between the TII cells and CRC clinical variables. First, we merged the TII cell distribution with the CRC clinical data and then conducted a Wilcoxon rank-sum test to compare between two groups. A Kruskal-Wallis test was used to compare three or more groups, and the results were illustrated using box plots. The strongly associated plots (*P* value < 0.05) were selected via ggalluvial package, and we presented the significant correlations between the TII cells and clinical variables using sankey plots.

### 2.4. Assessment of the Association between hub-ARP Signatures and TII Cells

Given the important roles of TII cells and autophagy in CRC, we estimated the association between hub-ARP signatures and TII cells to construct correlations between autophagy and TII and presented the results as a series of scatter plots. We selected the plots for which the statistical results were significant (*P* < 0.05) and presented the relationship between these signatures and TII cells via clusterProfiler, GOplot, tidyverse, data.table, ggraph, and tidygraph packages. In this cycle plot, the node size represents the |log *P* value| which was normalized to a range of 0.5-80.

### 2.5. Statistical Analysis

All data were used to determine the independent prognostic factors that could predict patient survival status with R package (R software version 3.5.2). GraphPad Prism 8.0 software was used to create plot graphs containing the Kaplan-Meier survival curve. All statistical analyses were performed using IBM SPSS 25.0 program. Student's *t*-test (for equal variances) was performed to the analyzed data, and a *P* value (two-sided test) less than 0.05 was considered to be significant with the purpose of ensuring the reliability of the results.

## 3. Results

### 3.1. The Characteristics of the Autophagy in CRC and Clinical Pathological Factors

**Figure 1:**
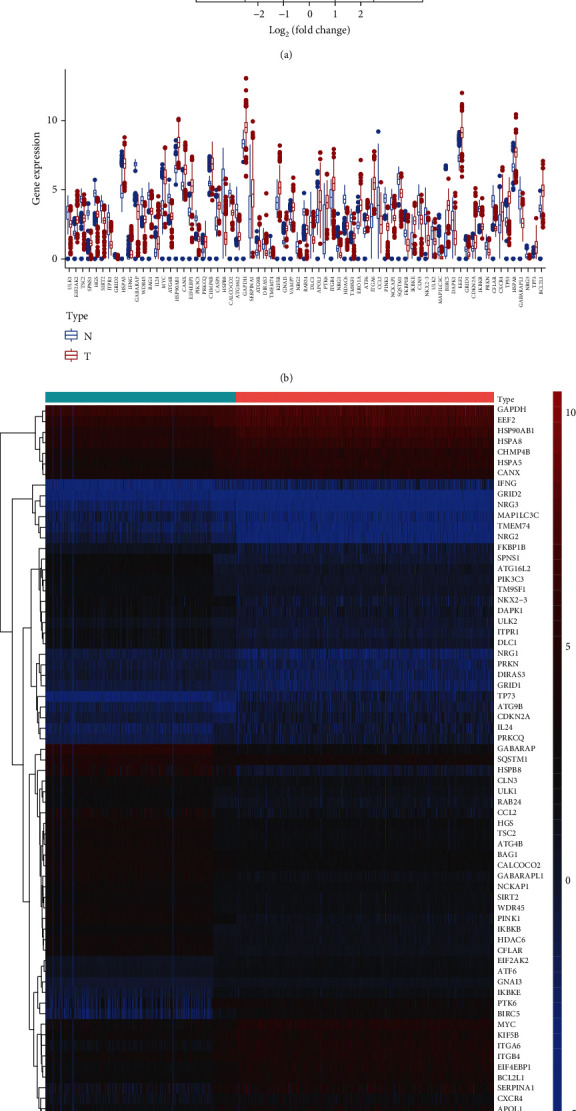
Identification of hub autophagy signature in breast cancer. (a) Volcano plot was drawn to show the differentially expressed genes in CRC versus normal samples. (b, c) The DEG expression in CRC versus normal samples was illustrated by box and heat map plots, respectively.

The gene expression profiles of 898 CRC samples were downloaded from TCGA and GTEx database, which included 473 tumor and 425 normal samples. Based on the clinical data, we excluded patients with incomplete symptom data as described in Table 1 (*n* = 452). Merging the CRC DEGs with a set of 232 autophagy-associated genes resulted in an overlap of 207 CAR genes (89.22% of the total). With a cutoff FDR value < 0.05, ∣logFC | >1, we screened 72 CAR DEGs, as illustrated in a volcano plot (Figure 1(a)), in which the blue dots represent downregulated gene probes and the red dots represent upregulated gene probes. The expression of the CAR DEGs is shown in Figures 1(b) and 1(c), which illustrates the comparison of the CAR DEGs between cancer and normal samples. To evaluate the value of these CAR DEGs in the pathogenesis of CRC, we conducted a series of functional enrichment analyses (Figure s1). According to the GO functional enrichment results (Figures s1A and s1B), these biosignatures participate in macroautophagy, release of cytochrome c from mitochondria [11], process utilizing autophagic mechanism, and autophagy. These biosignatures also play a role in the KEGG pathway in CRC autophagy (Figures s1C and s1D), p53 signaling pathway [12], apoptosis [13], EGFR tyrosine kinase inhibitor resistance [14], and the ErbB signaling pathway. These results suggest that the screened DEGs can be considered as hub signatures which participate in CRC-associated autophagy.

**Figure 2:**
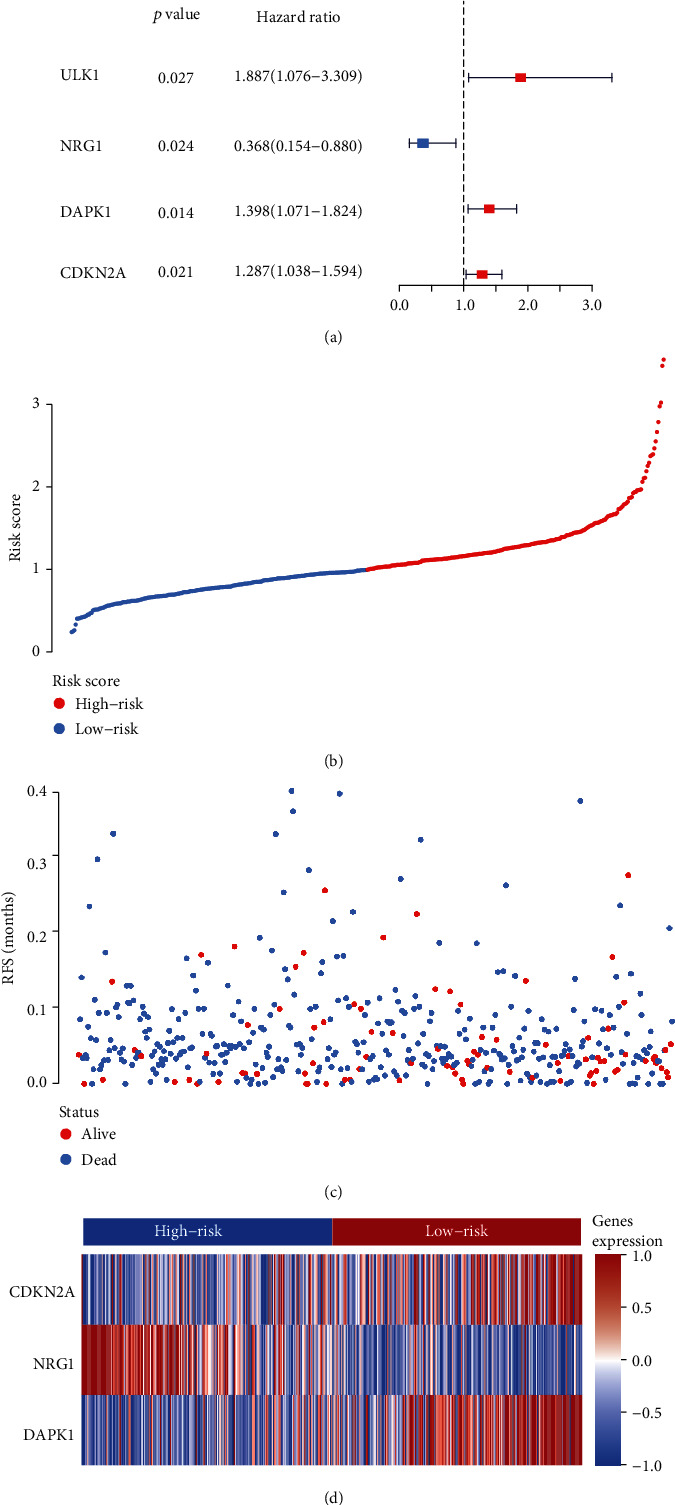
Identification of CRC-autophagy prognosis signatures and construction of a prognostic risk score system in CRC. (a) Forest plot visualizing hazard ratios of the expression level of the hub autophagy DEGs by performing univariate Cox regression analysis. (b) Patients' survival data based on risk score, (c) risk score's distribution curve, and (d) heat map of the PSI value of each prognostic signature.

To determine the association between the incidence and prognosis of CRC, perl was used to merge the clinical data based on the expression level of the hub autophagy DEGs described above. A univariate Cox regression analysis was used to identify the potential prognostic autophagy genes according to *p*Filter = 0.05, and the prognostic features are exhibited in Figure 2(a). Next, a multivariate Cox analysis was performed and an ARP risk model was built according to the weight of the respective signature (Table 2). According to the ARP risk score, the CRC patients enrolled in the present study were classified into two groups consisting of 224 low-risk and 223 high-risk patients. Figures 2(b) and 2(c) show that the high-risk group exhibited a higher survival risk. The hub signature expression data of the two groups are shown in Figure 2(d).

**Figure 3:**
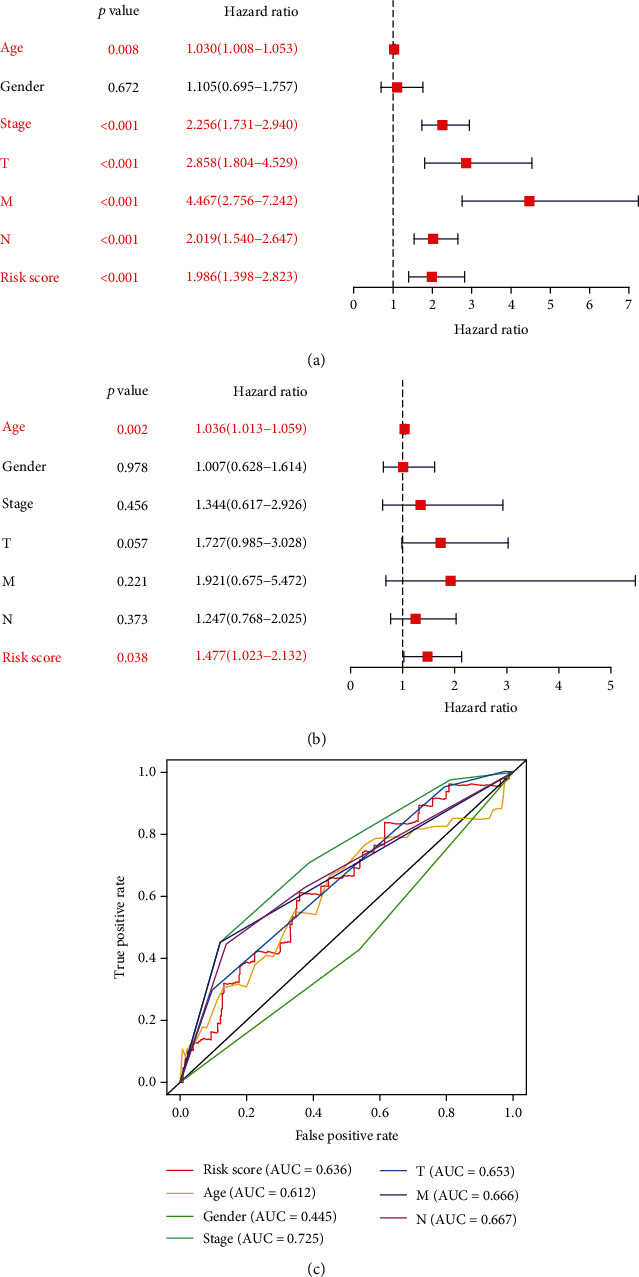
Forest plot visualizing hazard ratios of significantly survival-related clinical pathological parameters including age, gender, cancer stage, pathological stage T, M, and N, and risk score by performing (a) univariate and (b) multivariate survival analyses; and (c) an ROC plot was used to evaluate the ARP risk model regarding these different clinical pathological factors.

**Figure 4:**
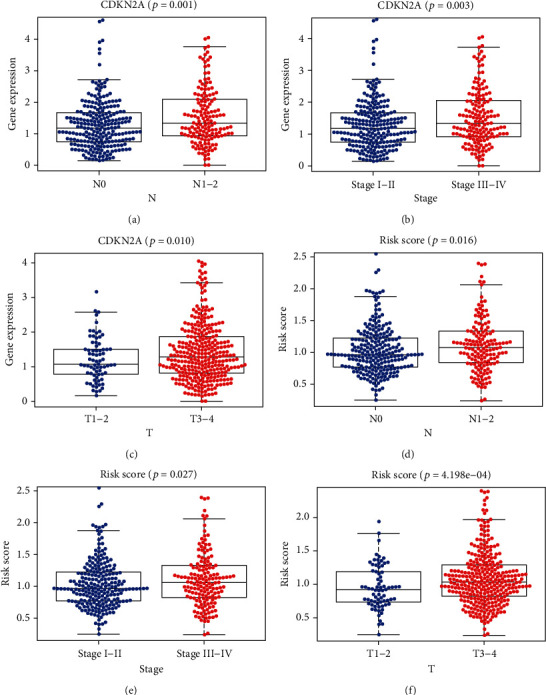
The close correlation between the ARP hub signatures and CRC clinical pathological factors (*p*Filter = 0.05).

To verify the predictive ability of this ARP risk model, univariate and multivariate survival analyses were used to evaluate the predictive ability of different clinical pathological factors, including age, gender, cancer stage, and pathological stage T, M, and N. The results of the univariate survival tests (Figure 3(a)) indicated that except for gender (*P* = 0.672), age (*P* = 0.008), cancer stage, and pathological stage T, M, and N were closely correlated with the prognosis of CRC patients (*P* < 0.001). Additionally, based on the multivariate survival analysis results (see Figure 3(b)), age and rescore were the only independent prognostic indicators with significant differences (*P* < 0.001). An ROC plot was used to evaluate the ARP risk model regarding the different clinical pathological factors. As shown in Figure s3C, the area under the curve (AUC) ranged from 0.613 (age) to 0.726 (stage), except gender, which exhibited a poor predictive ability (AUC = 0.446). Finally, we estimated the correlation between the ARP hub signatures and CRC clinical pathological factors by *p*Filter = 0.05. As shown in Figure 4, cancer stage, stage N, and stage T were closely associated with risk score and CDKN2A.

### 3.2. The Characteristics of the CRC Immune Microenvironment and Correlation between TII Cells and Clinical Pathological Factors

**Figure 5:**
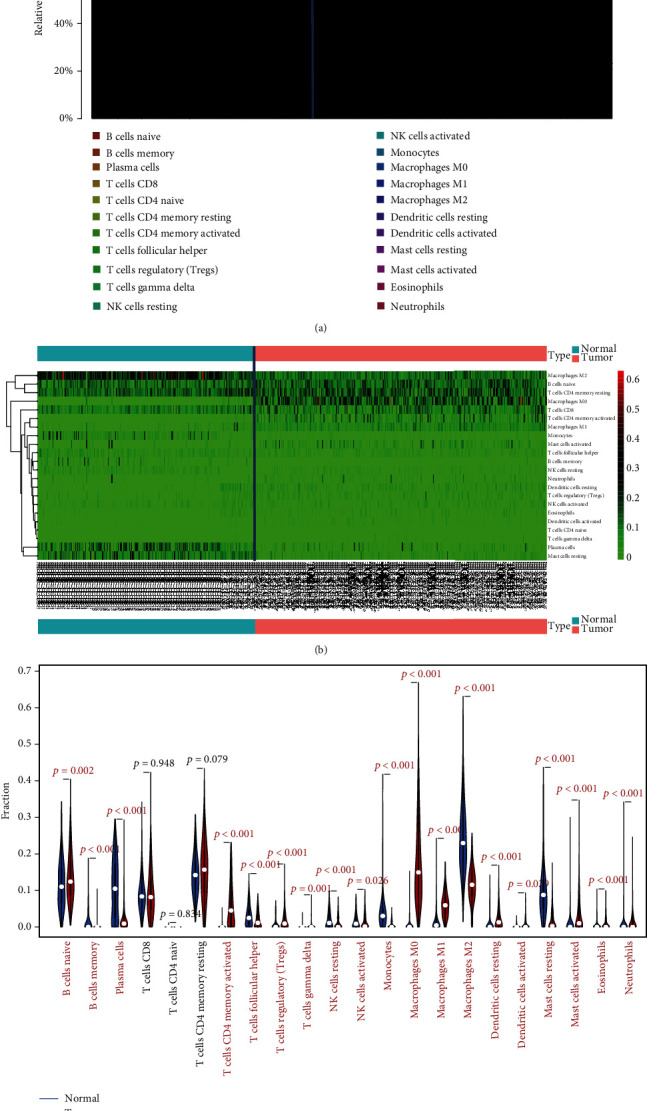
The distribution of 22 different types of TII cell in 514 CRC samples via CIBERSORT are shown by bar plots (a, b), and the distributional difference of the TII cells between the normal and tumor samples is illustrated in (c).

The TII profiles of the CRC patients were obtained from TCGA database, and after excluding samples with insufficient clinical data, we obtained 22 types of TII cell distribution in 898 samples via CIBERSORT (Figures s4A and s4B), including 425 normal samples and 473 tumor samples. The correlation among the CRC-TII cells is illustrated in Figure s2. Moreover, the distributional difference of the TII cells between the normal and tumor samples is illustrated in Figure 5. These results indicate that compared with the normal tissue, the tumor tissue exhibited a higher proportion of B cell naïve (*P* = 0.002), T cell CD4 memory activated (*P* < 0.01), T cell regulatory (Tregs) (*P* < 0.01), macrophage M0 (*P* < 0.01), macrophage M1 (*P* < 0.01), dendritic cell resting (*P* < 0.01), dendritic cell activated (*P* = 0.029), mast cell activated (*P* < 0.01), and eosinophil neutrophils (*P* < 0.01) and a lower proportion of B cell memory (*P* < 0.01), plasma cells (*P* < 0.01), T cell follicular helper plasma cells (*P* < 0.01), T cell gamma delta plasma cells (*P* < 0.01), NK cell resting (*P* < 0.01), NK cell activated (*P* = 0.026), monocytes (*P* < 0.01), macrophage M2 (*P* < 0.01), and mast cell resting (*P* < 0.01). Since naïve CD4 T cells were checked in only nine cases, these cells were not included in the analysis.

**Figure 6:**
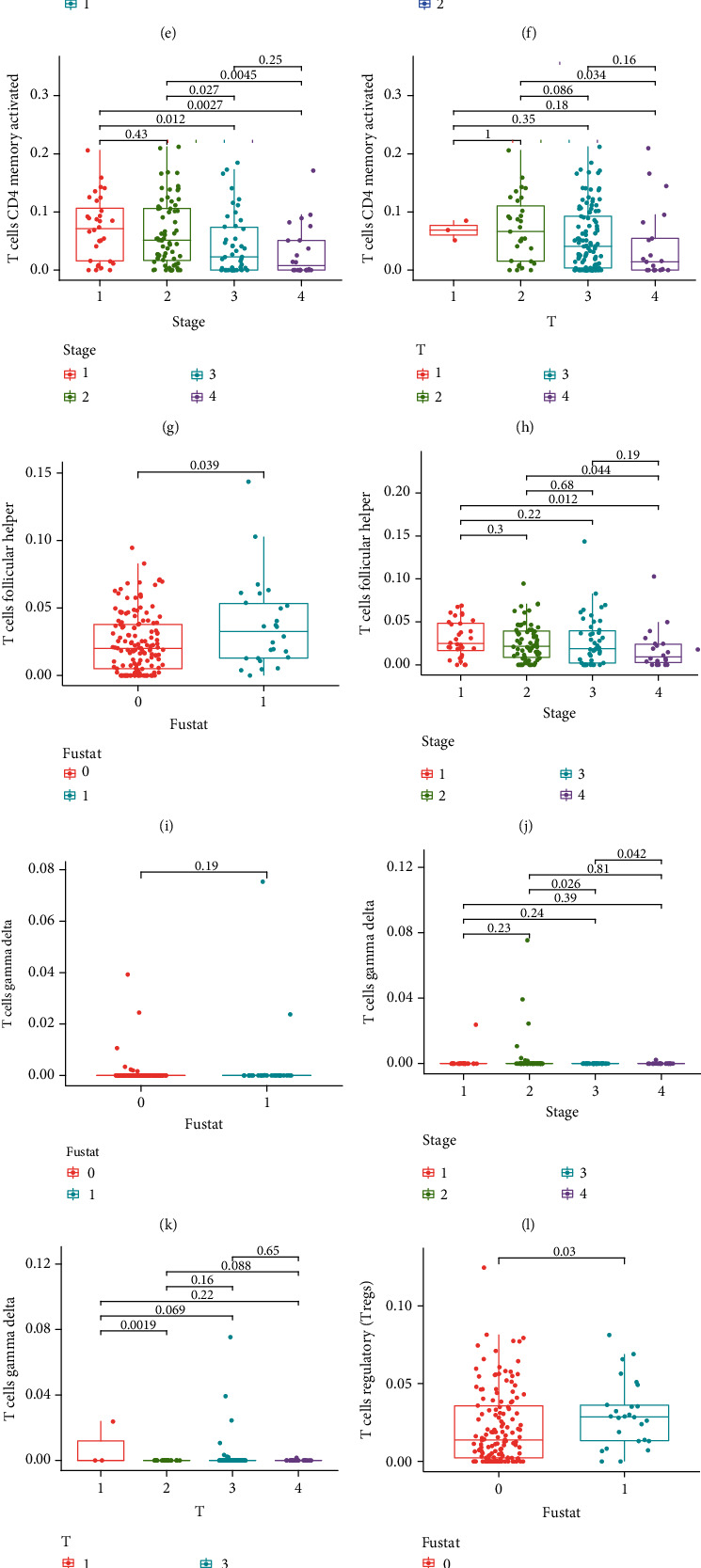
The significant correlation between the CRC immune microenvironment and clinical specificity pathological factors (*p*Filter = 0.05) is shown in (a–o), and (p) illustrates the association between 7 hub-TII cells and CRC clinical pathological factors.

Next, we merged the immune cell distribution with the CRC clinical data and further evaluated the correlation between the CRC immune microenvironment and clinical specificity (Figures s3–s8). After screening the plots that exhibited a significant correlation by *p*Filter = 0.05 (Figures 6(a)–6(o)), we analyzed the association between 7 hub-TII cells and CRC clinical pathological factors (Figure 6(p)). The results indicated that activated memory CD4 T cells exhibited the highest clinical response, which included cancer stage and stage T, N, and M. Additionally, six immune cells were closely associated with cancer stage, including regulatory T cells (Tregs), gamma delta T cells, follicular helper T cells, activated memory CD4 T cells, activated NK cells, and resting dendritic cells. Thus, these cells can be used for the clinical evaluation of CRC.

### 3.3. The Relationship between Autophagy and the TII Microenvironment of CRC

**Figure 7:**
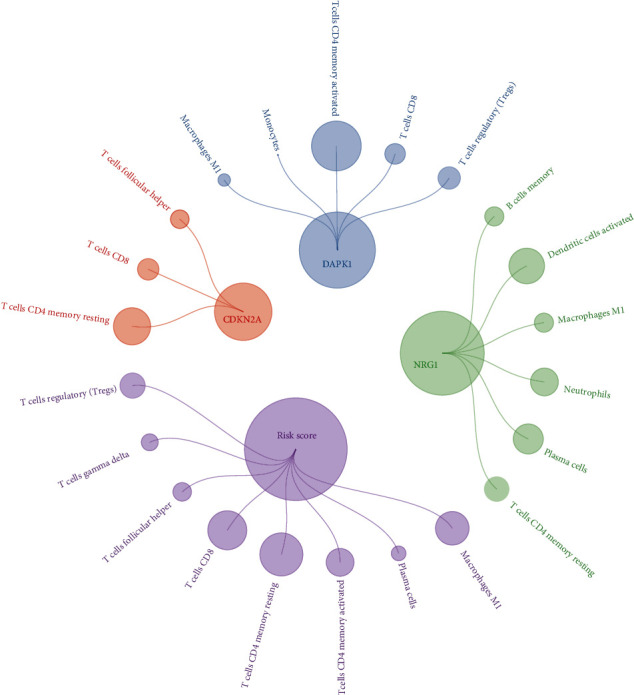
A circle plot exhibited the correlation of six ARP signatures and risk score with 19 types of TII cell distribution (*p*Filter = 0.05). The nod size represents the |log *P* value| and normalized to a range of 0.5-80, where red represented the positive correlation while green represented the negative correlation.

It has been reported that in the tumor microenvironment, abnormal TII cells are accompanied by an abnormal autophagy phenotype [15]. Briefly, the TII of CRC can affect the expression of a series of autophagy-related signatures. In the present study, we built up the association between the ARP hub signature expression, risk scores, and TII cell distribution (Figures s9–s12). We evaluated the correlation of three ARP signatures and risk score with 21 types of TII cell distribution. In the following step, excluding the plots that *P* > 0.05, we demonstrated the result in Table 3 and illustrated it by a circle plot (Figure 7), where the nod size represents the log_10_*P* value. According to the results of correlation analysis: (1) 12 TII cells, including monocytes, macrophage M1, plasma cells, T cell gamma delta, T cell follicular helper, B cell memory, T cell CD8, T cell regulatory (Tregs), T cell CD4 memory resting, neutrophils, T cell CD4 memory activated, and dendritic cell activated exhibited a close correlation to ARP hub signatures; (2) compared with the other cells, macrophage M1 exhibited the highest correlation with DAPK1 (↑, represents positive correlation), NRG (↓, represents negative correlation), risk score(↑) (*P* < 0.05). T cells CD4 memory resting are highly correlation with CDKN2A (↓), NRG1 (↑), and risk score (↓) (*P* < 0.05); and T cell CD8 exhibited the highest correlation with CDKN2A (↑), DAPK1 (↑), and risk score (↑) (*P* < 0.05). In contrast, B cell memory, dendritic cell activated, monocytes, neutrophils, and T cell gamma delta were only associated with the expression NRG1 (↓), NRG1 (↑), DAPK1 (↓), NRG1 (↑), and risk score (↑) (*P* < 0.05); (3) in all ARP three signatures, NRG1 exhibited the most correlation to the fractions of TII cells, including B cell memory (↓), dendritic cell activated (↑), macrophage M1 (↓), neutrophils (↑), plasma cells (↑), and T cell CD4 memory resting (↑) as the ARP risk score is closely correlated with 8 TII cells, including macrophage M1 (↑), plasma cells (↓), T cell CD4 memory activated (↓), T cell CD4 memory resting (↓), T cell CD8 (↑), T cell follicular helper (↑), T cell gamma delta (↑), and T cell regulatory (Tregs) (↑) (*P* < 0.05); (4) compared with the other correlation, NRG1 and plasma cells (↑), risk score and macrophage M1 (↑), NRG1 and dendritic cell activated (↑), CDKN2A and T cell CD4 memory resting (↓), risk score and T cell CD8 (↑), risk score and T cell CD4 memory resting (↓), and DAPK1 and T cell CD4 memory activated (↓) exhibited a stronger association (*P* < 0.0001).

## 4. Discussion

CRC is the fourth most common malignancy [16] and leading cause of cancer-related death [1] worldwide. According to Siegel et al. [17], there were 148,000 diagnosed CRC patients and 53,000 deaths in the US in 2020. Irrespective of age, the male morbidity rate of CRC is 44.4/100,000 and 34.4/100,000 in females [17]. Since mechanistic studies may represent an important approach to clinical CRC treatment and prognosis, several studies have concentrated on the pathogenesis and progression of CRC. This research has revealed that the pathogenesis and development of CRC are a complex process that involves multiple pathways and phenotypes [18]. Moreover, increasing evidence has demonstrated that autophagy and TII play an important role throughout the entire CRC process [19, 20]. As a biological phenomenon, autophagy is ubiquitous in eucaryotes and lysosomes are used to degrade damaged organelles and bio-macromolecules [21]. The role of autophagy in cancer has been found to be divergent [22]. First, autophagy may exhibit tumor-suppressing properties due to the maintenance of cell homeostasis via damage repair, removal of harmful substances, and support of gene stability, thereby inhibiting carcinogenesis. On the other hand, the autophagy of damaged cancer cells can allow the tumor tissue to obtain energy and nutrients for cellular growth, metabolism, and proliferation. Previous studies focusing on the genetic landscape in human cancer have demonstrated that [23, 24] autophagy overlaps with a series of cancer-related phenotypes, and crosstalk genes can be regarded as a hub of biosignatures which could provide a target for the clinical prediction and treatment of CRC.

Previous studies have shown that a deletion in autophagy-related genes results in the promotion of tumor inflammation [25, 26], whereas inhibiting autophagy stimulates the activation of CD8+ T cells [27]. Moreover, autophagy plays an important role in memory and effector memory T cell differentiation [28]. A deficiency of tumor cells to autophagy sensitivity could indicate a blockage of immune checkpoints, which results in the resistance of tumor cells to therapy [29]. These studies demonstrate that autophagy participates deeply in the entire processes within a tumor and plays a critical role in immune escape or drug resistance of a tumor.

In the immune microenvironment of the normal tissue, the immune system can recognize tumor-associated antigens, secrete immune effector molecules caused by the activation of effector immune cells, and suppress tumor growth and induce apoptosis of the tumor cells [30]. However, a cancer immune microenvironment consisting of tumor-infiltrating lymphocytes (TILs), peripheral vessels, and fibrocytes which aggregate immunosuppressive cells and cytokines promotes immunosuppression and is associated with immune escape by cancer cells [31]. For example, B7-H4 expressed by antigen-presenting cells may bind to the B7-H4 ligand on donor T cells, block the proliferation and differentiation of T cells, and secrete immunosuppressive factors (e.g., IL10 and TGF-*β*) [32, 33]. Arginase-1 targets the degradation of L(+)-arginine, thus inducing functional unresponsiveness in T cells via the inhibition of IFN-*γ* generation by CD8^+^ T cells [34–36].

Based on the findings of these studies, we inferred that the correlation between autophagy and TII may be considered to be a potential signature for the prognosis and immune-therapeutic targets in CRC patients. With the estimate of TII level in the CRC immune microenvironment and the expression of APR genes, we established an autophagy-TII risk model and evaluated the autophagy-TII signatures to obtain a series of precise targets for immunotherapy in the future.

Based on TCGA database, the present study identified 207 autophagy signatures and 72 DEGs related to CRC. According to the results of the COX analysis, three signatures (CDKN2A, DAPK1, and NRG1) were selected as the ARP signatures and an ARP risk model was established for CRC. Death-associated protein kinase 1 (DAPK1) belongs to the DAPK family. CDKN2A (multiple tumor suppressor l, MTS1 or P16) is a well-known classic tumor suppressor and the loss of p16 may be an early event in cancer progression [37]. Shima et al. summarized the functions of CDKN2A in colorectal cancers and confirmed that neither CDKN2A promoter methylation nor loss of CDKN2A (p16) was associated with colorectal cancer-specific mortality [38]. In Kong et al.'s research [39], CDKN2A could be a reliable drug target of fenofibrate for colon cancer therapy. As a serine/threonine protein kinase, DAPK1 is considered to be a cancer suppressor gene, which is regulated by calmodulin (CaM) and is a positive regulator of IFN-*γ*-induced apoptosis [40]. The hypermethylation of the CpG island in the DAPK1 promoter region was examined in a series of tumor tissue and cancer cells, such as cervical cancer [41], gliomas [42], and colorectal cancer [43]. Moreover, several studies indicate that DAPK1 is involved in tumor invasion and metastasis, as the loss of DAPK1 enhanced tumor budding and increased the invasion capacity [44, 45]. The increased expression of DAPK1 could suppress the metastasis of lung cancer cells [46]. DAPK1 expression induced autophagy and apoptotic activity in various cancers [47] by causing autophagic cell death via reducing the interaction between Beclin-1, Bcl-2, and Bcl-X_L_ [48]. NRG1 gene was considered as an important signature of Hirschsprung disease [49–51]. It can be speculated that NRG1 participate in a series of colon disease; in the tumor microenvironment, NRG1 could promote antiandrogen resistance in prostate cancer [52]. NRG1 gene fusion takes part in the progression of breast cancer [53] and lung adenocarcinoma [54].

As previously mentioned, autophagy plays a positive role in the course of immune killing of tumor cells [25, 26]. In pancreatic cancer [55], triple-negative breast cancer [56], and non-small-cell lung cancer [57] immune treatment, the promotion of autophagy results in higher anticancer activity of immune cells. In the present study, we utilized CIBERSORT to display the immune landscape of the infiltration of 22 immune cells in CRC and further obtained the correlation between these immune cells and CRC clinical features. Our results show that seven types of immune cells inferred the cancer stage, stage T, stage N, and stage M, in which activated memory CD4 T cells contributed the greatest relevance, including cancer stage, stage T, stage N, and stage M. Previous research indicates that [58] Tax2 is linked to memory CD4 T cells and autophagy in adult T cell leukemia/lymphoma (ATL). However, limited research has established a correlation between the immune response of activated memory CD4 T cell and autophagy in CRC. Clearly, compared with the other immune cell types, activated memory CD4 T cells can be considered the most important immune cell type during CRC pathogenesis and progression. On the other hand, the level of immune cells was closely correlated with cancer stage, which infers that the TII level is closely correlated with CRC severity. To further disentangle the direct correlation between autophagy and TII, we performed a series of correlation analyses between AUT-related signatures and TII cells.

Based on the results of this study, these three signatures are strongly correlated with the TII level in CRC. 12 immune cell types are closely related to the signatures selected, because neutrophils could enhance the migratory ability of circulating tumor cells (CTCs) via the release of various special messenger substances (e.g., cytokines) [59]. The present study demonstrates that neutrophils are involved in the process of CRC initiation, development, and metastasis through autophagy. Moreover, the AUT risk score is closely associated with eight immune cell types (Table 3). Clearly, the degree of these immune cell types in the CRC immune microenvironment was dominated by autophagy. Finally, in all three AUT signatures, NRG1 exhibited a strong correlation with TII, both quantitatively (six types) and qualitatively (dendritic cell activated and plasma cells). These results suggest that NRG1 is the most important biosignature crosstalk in both autophagy and TII of CRC.

Taken together, the present study carried out a Cox multivariate analysis to develop the risk score model. We then identified and validated the AUT-related signatures according to an AUT risk scoring system and identified a connection between the AUT risk and prognosis of CRC patients. We conducted a correlation analysis according to the survival outcome of the patients, and the correlation between TII and AUT was evaluated. Finally, we constructed the association between five AUT-related signatures and 20 TII cells. These selected genes can also provide novel signatures that target both the autophagy signaling pathway and immune regulation for CRC immunotherapy.

Compared with the traditional study of CRC biomarkers, our study analyzed a large number of clinical samples and focused on the characteristics of autophagy in TII. We investigated the relationship between the regulation of AUT-related genes to TII and the association of autophagy and TII cells with the incidence and prognosis of CRC.

The present study utilized large TCGA database cohorts to elucidate and validate relevant signatures of CRC risk, as well as further evaluate their clinical characteristics. Next, with CIBERSORT tools, we established the AUT-TII predication model. This approach reduced the workload and thereby the screening time; however, the results obtained in this study were completely dependent on fitting and the accuracy requires further verification with a prospective cohort. In addition, the signatures obtained need to be further investigated in clinical studies with a greater number of patients. Therefore, these findings confirm their biofunction and provide an experimental basis for clinical treatment.

With the methods used in this research, we can utilize public databases to systematically study the relationship between autophagy and TII in CRC. Through the TII cell landscape and autophagy characteristics, we evaluated the correlation between five AUT signatures with the TII cell profile, which further provides novel therapeutic targets for CRC treatment. In addition to CRC, our study can provide novel methods for further potential research on other cancer-related studies.

## Figures and Tables

**Table 1:** Baseline characteristics of 452 CRC patients included in this study.

Variables	Count	Percentage (%)
Age (mean ± SD)	67.26 ± 13.02	
Follow-up (y)	2.05 ± 1.98	
Status		
Alive	88	19.47
Dead	364	80.53
Gender		
Male	238	52.65
Female	214	47.34
AJCC-T		
T1	10	2.21
T2	77	17.04
T3	308	68.14
T4	56	12.39
Tis	1	0.22
AJCC-N		
N0	269	59.51
N1	103	22.79
N2	80	17.70
AJCC-M		
M0	334	73.89
M1	62	13.72
MX	49	10.84
Unknown	7	1.55
Pathological stage		
I	76	16.81
II	178	39.38
III	125	27.65
IV	62	13.72
Unknown	11	2.43
Grade		
G1	—	—
G2	—	—
G3	—	—
G4	—	—
Unknown	452	100.00

Abbreviations: AJCC: American Joint Committee on Cancer.

**Table 2:** The detailed information of AUT signatures related to overall survival in CRC patients (*n* = 447).

id	coef	HR	HR.95L	HR.95H	*P* value
NRG1	-0.90036	0.406423	0.168212	0.981974	0.045459
DAPK1	0.264768	1.303129	1.002567	1.693797	0.047802
CDKN2A	0.21178	1.235876	1.001489	1.525118	0.048401

**Table 3:** The association between ARP hub signature expression and TII cell distribution in CRC patients (*P* < 0.05).

Gene	Cell	*P*	Cor	Correlation
CDKN2A	T cell CD4 memory resting	0.00004307	-0.192	Negative
CDKN2A	T cell CD8	0.005	0.132	Positive
CDKN2A	T cell follicular helper	0.009	0.124	Positive
DAPK1	Macrophage M1	0.023	0.107	Positive
DAPK1	Monocytes	0.046	-0.094	Negative
DAPK1	T cell CD4 memory activated	1.452*E* − 07	-0.246	Negative
DAPK1	T cell CD8	0.006	0.129	Positive
DAPK1	T cell regulatory (Tregs)	0.004	0.137	Positive
NRG1	B cell memory	0.008	-0.125	Negative
NRG1	Dendritic cell activated	0.00007818	0.186	Positive
NRG1	Macrophage M1	0.008	-0.126	Negative
NRG1	Neutrophils	0.001	0.155	Positive
NRG1	Plasma cells	0.0005104	0.164	Positive
NRG1	T cell CD4 memory resting	0.002	0.149	Positive
Risk score	Macrophage M1	0.0001605	0.178	Positive
Risk score	Plasma cells	0.017	-0.113	Negative
Risk score	T cell CD4 memory activated	0.001	-0.151	Negative
Risk score	T cell CD4 memory resting	0.000003698	-0.217	Negative
Risk score	T cell CD8	0.00002072	0.2	Positive
Risk score	T cell follicular helper	0.009	0.124	Positive
Risk score	T cell gamma delta	0.012	0.119	Positive
Risk score	T cell regulatory (Tregs)	0.002	0.144	Positive

## Data Availability

The original data used to support the findings of this study was obtained from public databases (TCGA, GTEx, and Human Autophagy Database).

## Supplementary Materials

Supplementary Materials Figure s1: functional enrichment analysis results of differential autophagy genes in CRC. (A, B) Significantly enriched Gene Ontology terms of differential autophagy genes in CRC. BP: biological process; CC: cellular component; MF: molecular function. (C, D) Significantly enriched Kyoto Encyclopedia of Genes and Genomes pathways of differential autophagy genes in CRC. Figure s2: correlation between tumor-infiltrating immune cells where red represented the positive correlation while green represented the negative correlation in CRC. Figure s3: the correlation between the CRC immune microenvironment and fustat. Figure s4: the correlation between the CRC immune microenvironment and gender. Figure s5: the correlation between the CRC immune microenvironment and pathological stage M. Figure s6: the correlation between the CRC immune microenvironment and pathological stage N. Figure s7: the correlation between the CRC immune microenvironment and cancer stage. Figure s8: the correlation between the CRC immune microenvironment and pathological stage T. Figure s9: the correlation of DAPK1 with 21 types of TII cell distribution. Figure s10: the correlation of MAP1LC3C with 19 types of TII cell distribution. Figure s11: the correlation of PELP1 with 19 types of TII cell distribution. Figure s12: the correlation of risk score with 19 types of TII cell distribution.
