# Supplementary figures and images for: The Role of Autophagy in Tumor Immune Infiltration in Colorectal Cancer
Source: Anal Cell Pathol (Amst). 2022 Mar 14;2022:2055676. doi: 10.1155/2022/2055676 (PMC8938087; doi:10.1155/2022/2055676)

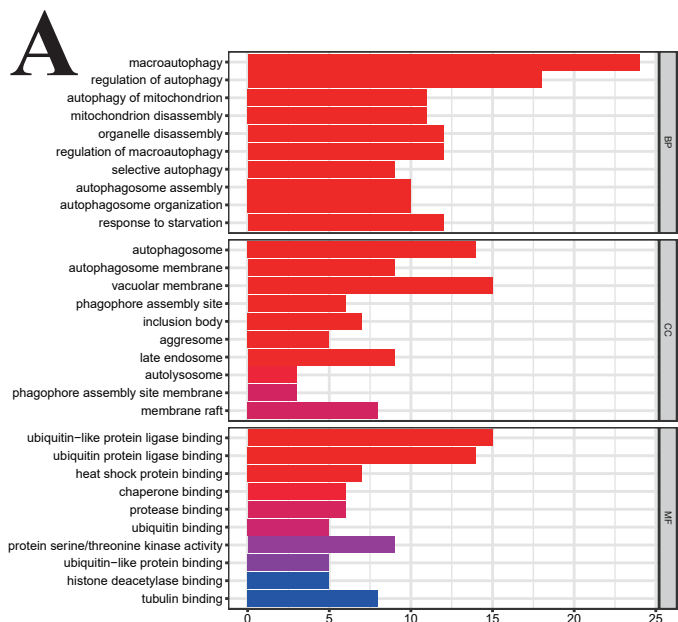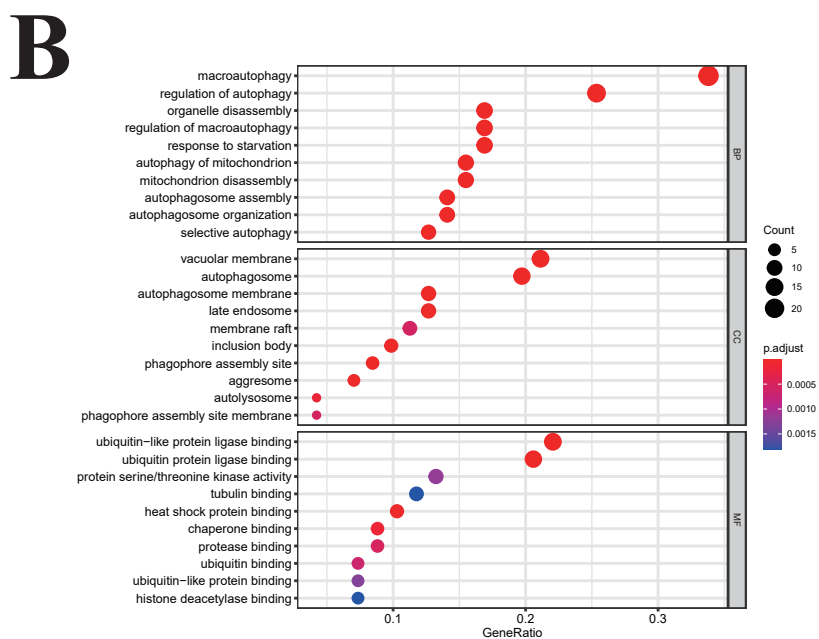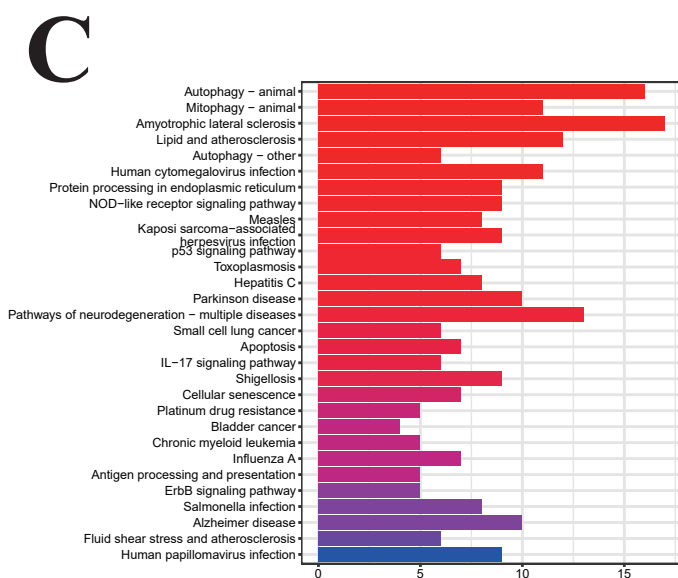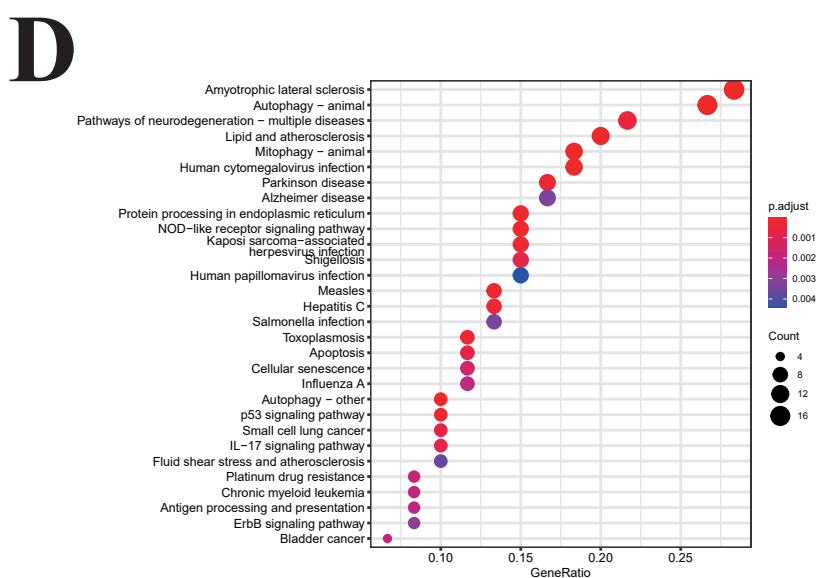

Supplement: Supplementary Materials — Figure s1: functional enrichment analysis results of differential autophagy genes in CRC. (A, B) Significantly enriched Gene Ontology terms of differential autophagy genes in CRC. BP: biological process; CC: cellular component; MF: molecular function. (C, D) Significantly enriched Kyoto Encyclopedia of Genes and Genomes pathways of differential autophagy genes in CRC. Figure s2: correlation between tumor-infiltrating immune cells where red represented the positive correlation while green represented the negative correlation in CRC. Figure s3: the correlation between the CRC immune microenvironment and fustat. Figure s4: the correlation between the CRC immune microenvironment and gender. Figure s5: the correlation between the CRC immune microenvironment and pathological stage M. Figure s6: the correlation between the CRC immune microenvironment and pathological stage N. Figure s7: the correlation between the CRC immune microenvironment and cancer stage. Figure s8: the correlation between the CRC immune microenvironment and pathological stage T. Figure s9: the correlation of DAPK1 with 21 types of TII cell distribution. Figure s10: the correlation of MAP1LC3C with 19 types of TII cell distribution. Figure s11: the correlation of PELP1 with 19 types of TII cell distribution. Figure s12: the correlation of risk score with 19 types of TII cell distribution. [file 2055676.f1.zip › 2055676.f1.pdf]

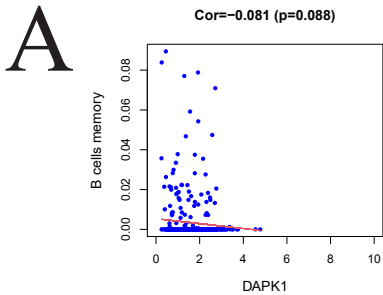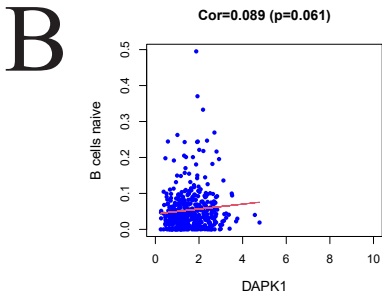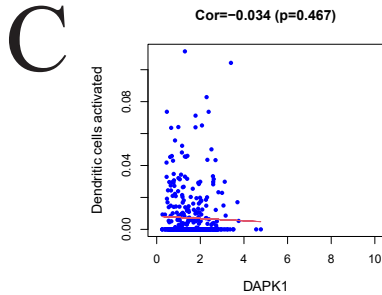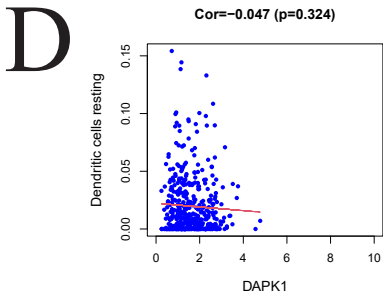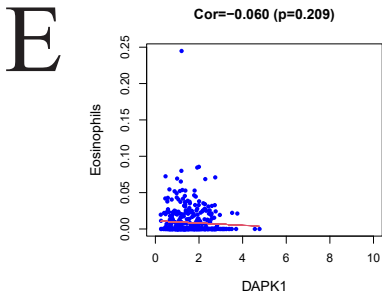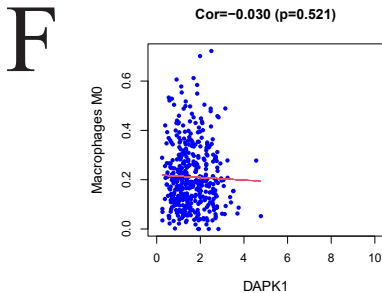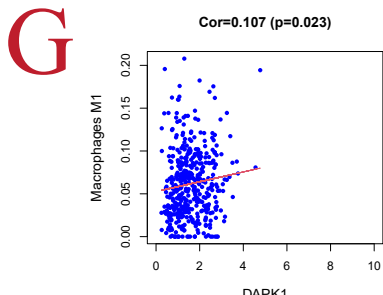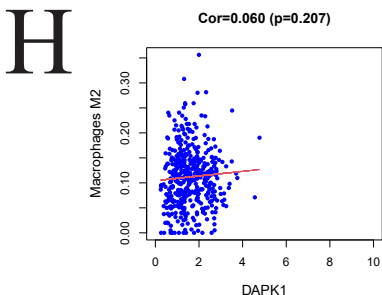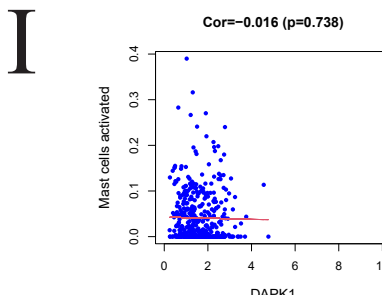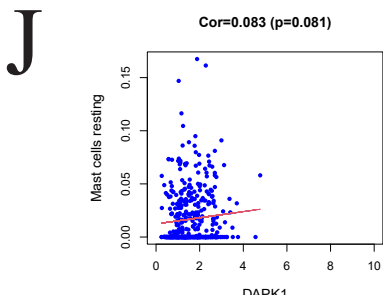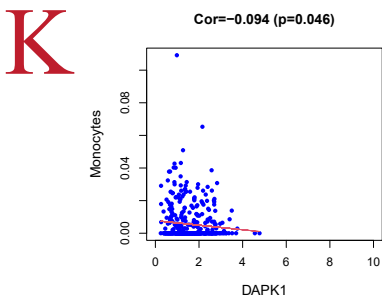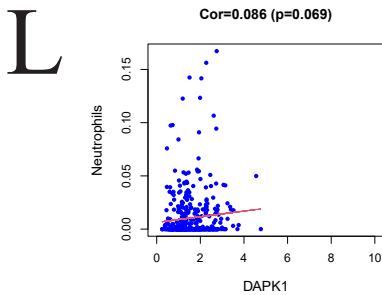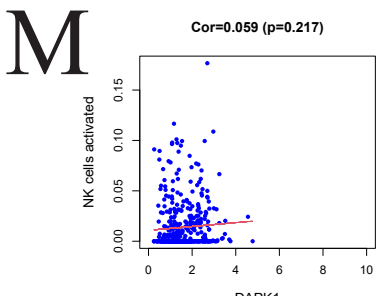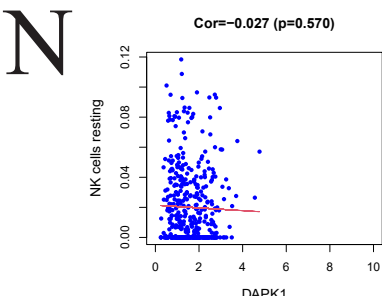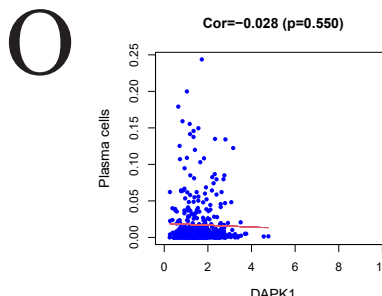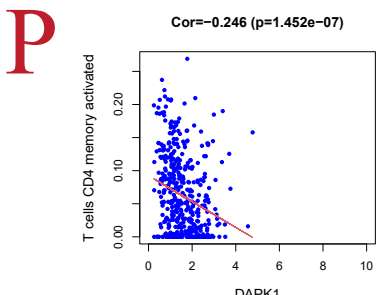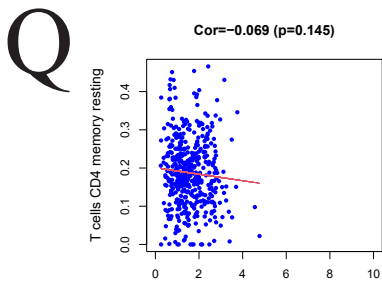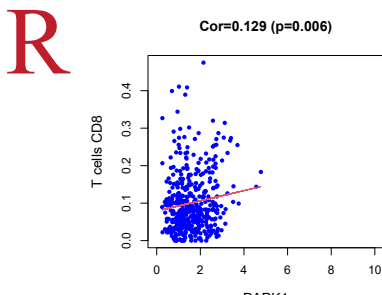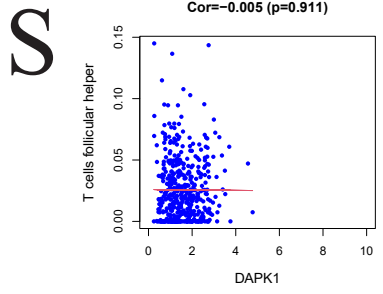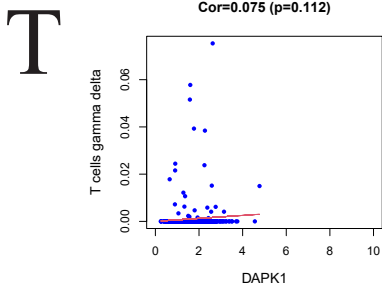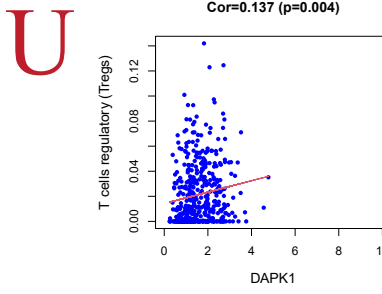

Supplement: Supplementary Materials — Figure s1: functional enrichment analysis results of differential autophagy genes in CRC. (A, B) Significantly enriched Gene Ontology terms of differential autophagy genes in CRC. BP: biological process; CC: cellular component; MF: molecular function. (C, D) Significantly enriched Kyoto Encyclopedia of Genes and Genomes pathways of differential autophagy genes in CRC. Figure s2: correlation between tumor-infiltrating immune cells where red represented the positive correlation while green represented the negative correlation in CRC. Figure s3: the correlation between the CRC immune microenvironment and fustat. Figure s4: the correlation between the CRC immune microenvironment and gender. Figure s5: the correlation between the CRC immune microenvironment and pathological stage M. Figure s6: the correlation between the CRC immune microenvironment and pathological stage N. Figure s7: the correlation between the CRC immune microenvironment and cancer stage. Figure s8: the correlation between the CRC immune microenvironment and pathological stage T. Figure s9: the correlation of DAPK1 with 21 types of TII cell distribution. Figure s10: the correlation of MAP1LC3C with 19 types of TII cell distribution. Figure s11: the correlation of PELP1 with 19 types of TII cell distribution. Figure s12: the correlation of risk score with 19 types of TII cell distribution. [file 2055676.f1.zip › 2055676.f10.pdf]

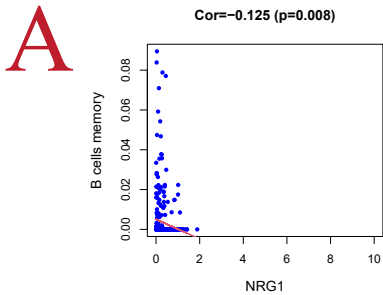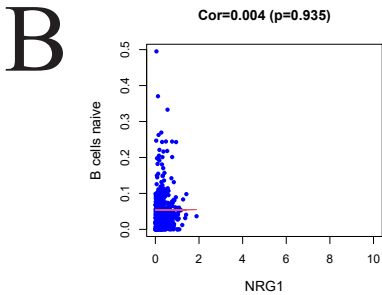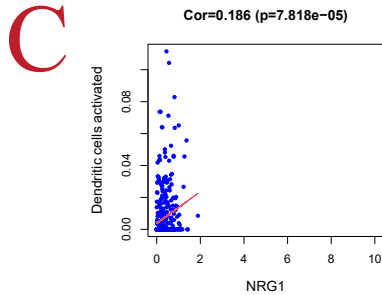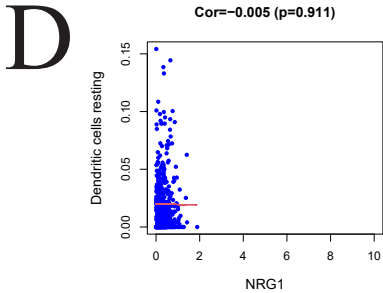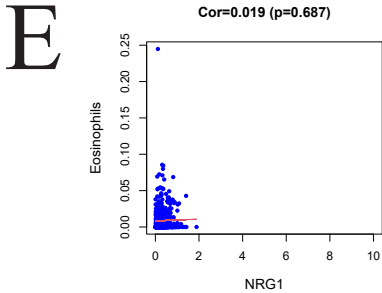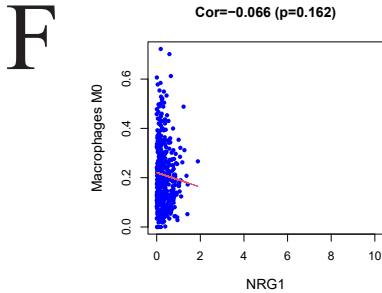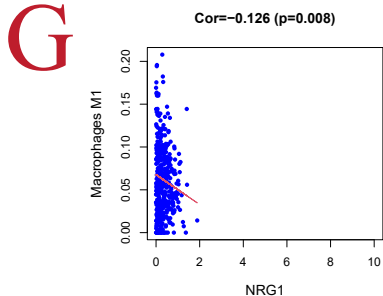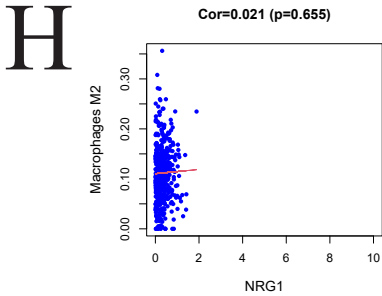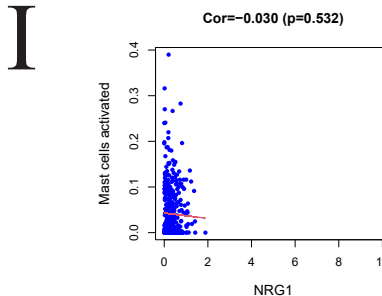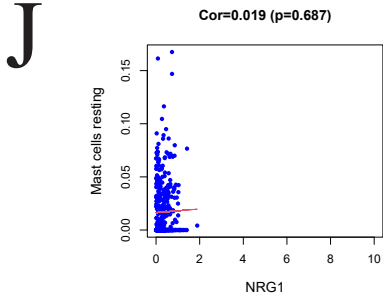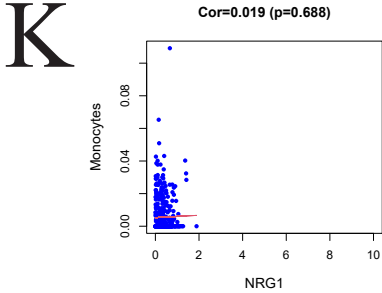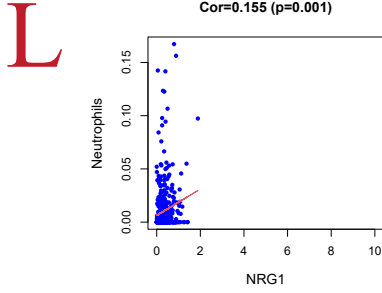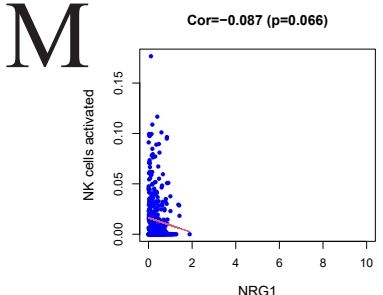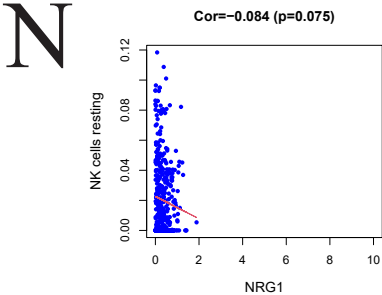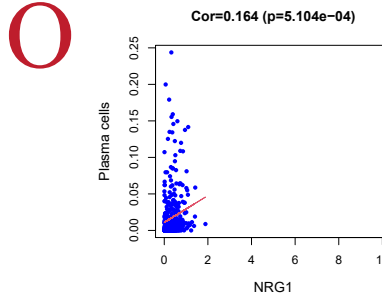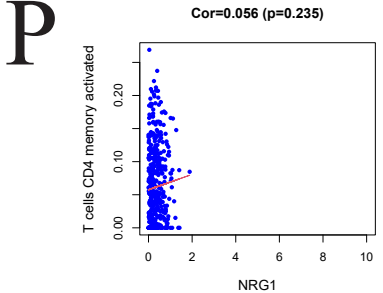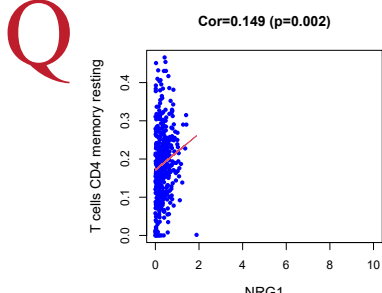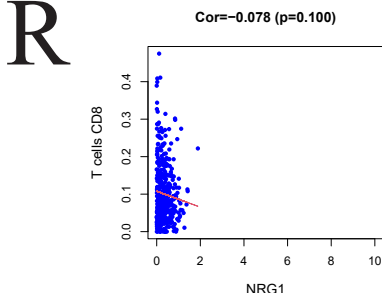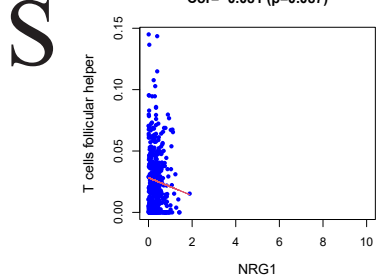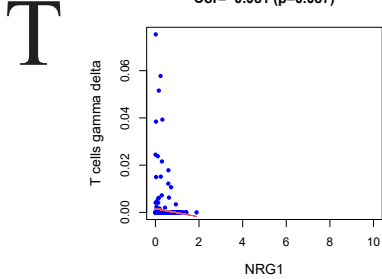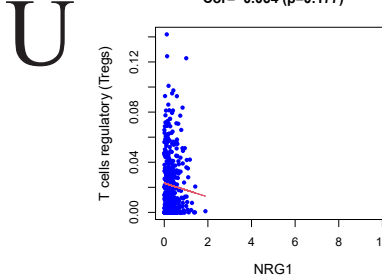

Supplement: Supplementary Materials — Figure s1: functional enrichment analysis results of differential autophagy genes in CRC. (A, B) Significantly enriched Gene Ontology terms of differential autophagy genes in CRC. BP: biological process; CC: cellular component; MF: molecular function. (C, D) Significantly enriched Kyoto Encyclopedia of Genes and Genomes pathways of differential autophagy genes in CRC. Figure s2: correlation between tumor-infiltrating immune cells where red represented the positive correlation while green represented the negative correlation in CRC. Figure s3: the correlation between the CRC immune microenvironment and fustat. Figure s4: the correlation between the CRC immune microenvironment and gender. Figure s5: the correlation between the CRC immune microenvironment and pathological stage M. Figure s6: the correlation between the CRC immune microenvironment and pathological stage N. Figure s7: the correlation between the CRC immune microenvironment and cancer stage. Figure s8: the correlation between the CRC immune microenvironment and pathological stage T. Figure s9: the correlation of DAPK1 with 21 types of TII cell distribution. Figure s10: the correlation of MAP1LC3C with 19 types of TII cell distribution. Figure s11: the correlation of PELP1 with 19 types of TII cell distribution. Figure s12: the correlation of risk score with 19 types of TII cell distribution. [file 2055676.f1.zip › 2055676.f11.pdf]

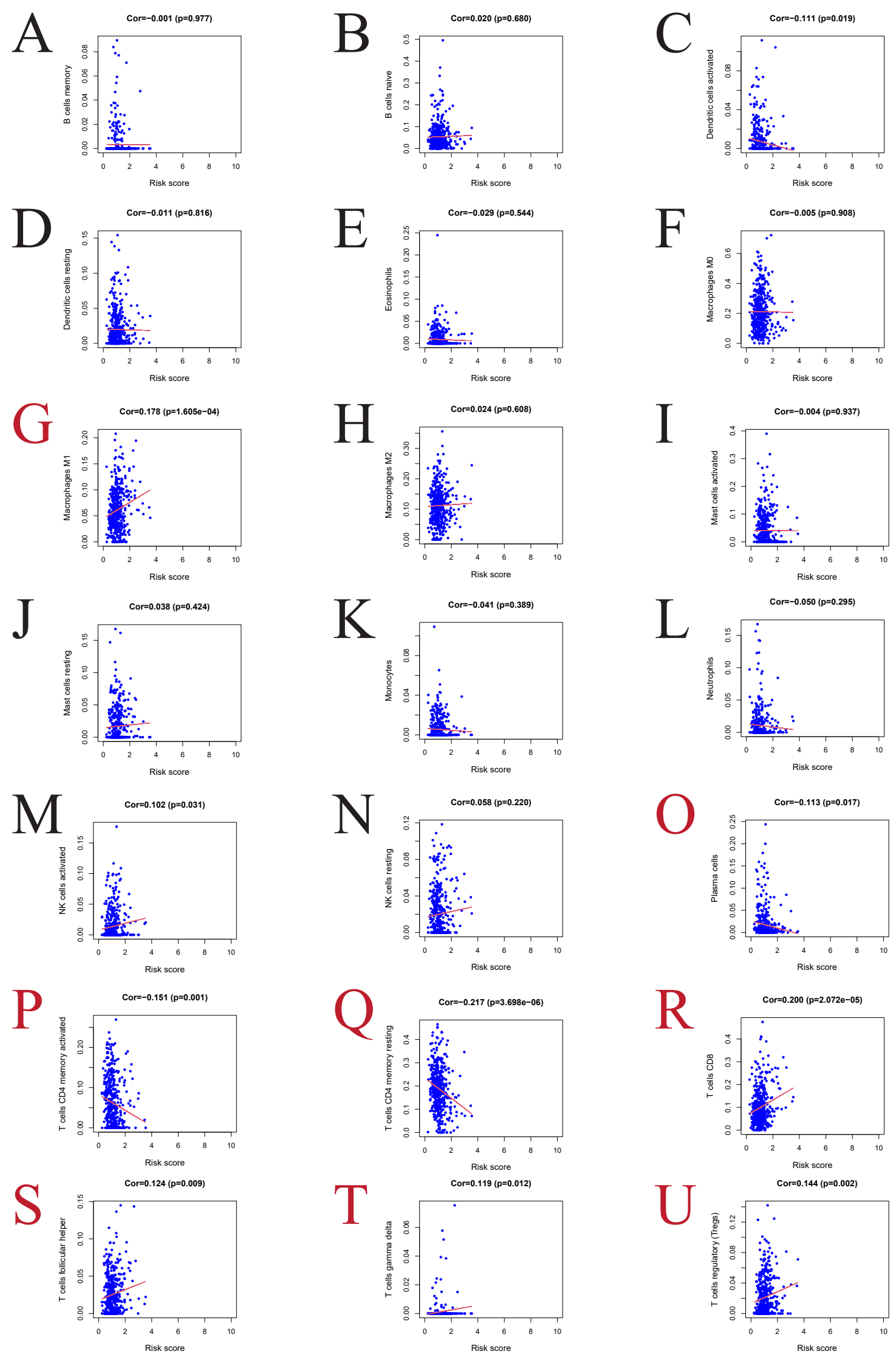

Supplement: Supplementary Materials — Figure s1: functional enrichment analysis results of differential autophagy genes in CRC. (A, B) Significantly enriched Gene Ontology terms of differential autophagy genes in CRC. BP: biological process; CC: cellular component; MF: molecular function. (C, D) Significantly enriched Kyoto Encyclopedia of Genes and Genomes pathways of differential autophagy genes in CRC. Figure s2: correlation between tumor-infiltrating immune cells where red represented the positive correlation while green represented the negative correlation in CRC. Figure s3: the correlation between the CRC immune microenvironment and fustat. Figure s4: the correlation between the CRC immune microenvironment and gender. Figure s5: the correlation between the CRC immune microenvironment and pathological stage M. Figure s6: the correlation between the CRC immune microenvironment and pathological stage N. Figure s7: the correlation between the CRC immune microenvironment and cancer stage. Figure s8: the correlation between the CRC immune microenvironment and pathological stage T. Figure s9: the correlation of DAPK1 with 21 types of TII cell distribution. Figure s10: the correlation of MAP1LC3C with 19 types of TII cell distribution. Figure s11: the correlation of PELP1 with 19 types of TII cell distribution. Figure s12: the correlation of risk score with 19 types of TII cell distribution. [file 2055676.f1.zip › 2055676.f12.pdf]

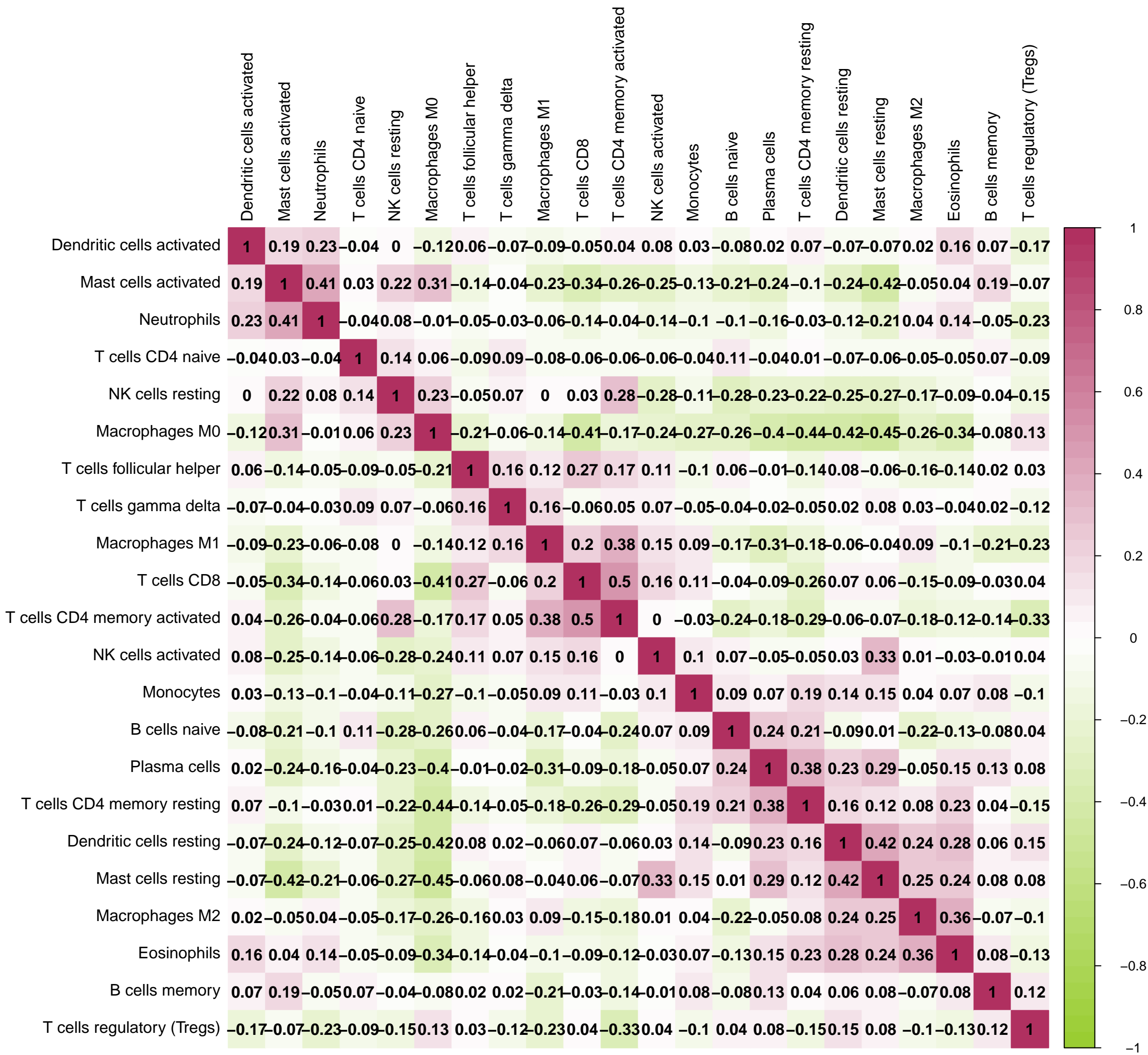

Supplement: Supplementary Materials — Figure s1: functional enrichment analysis results of differential autophagy genes in CRC. (A, B) Significantly enriched Gene Ontology terms of differential autophagy genes in CRC. BP: biological process; CC: cellular component; MF: molecular function. (C, D) Significantly enriched Kyoto Encyclopedia of Genes and Genomes pathways of differential autophagy genes in CRC. Figure s2: correlation between tumor-infiltrating immune cells where red represented the positive correlation while green represented the negative correlation in CRC. Figure s3: the correlation between the CRC immune microenvironment and fustat. Figure s4: the correlation between the CRC immune microenvironment and gender. Figure s5: the correlation between the CRC immune microenvironment and pathological stage M. Figure s6: the correlation between the CRC immune microenvironment and pathological stage N. Figure s7: the correlation between the CRC immune microenvironment and cancer stage. Figure s8: the correlation between the CRC immune microenvironment and pathological stage T. Figure s9: the correlation of DAPK1 with 21 types of TII cell distribution. Figure s10: the correlation of MAP1LC3C with 19 types of TII cell distribution. Figure s11: the correlation of PELP1 with 19 types of TII cell distribution. Figure s12: the correlation of risk score with 19 types of TII cell distribution. [file 2055676.f1.zip › 2055676.f2.pdf]

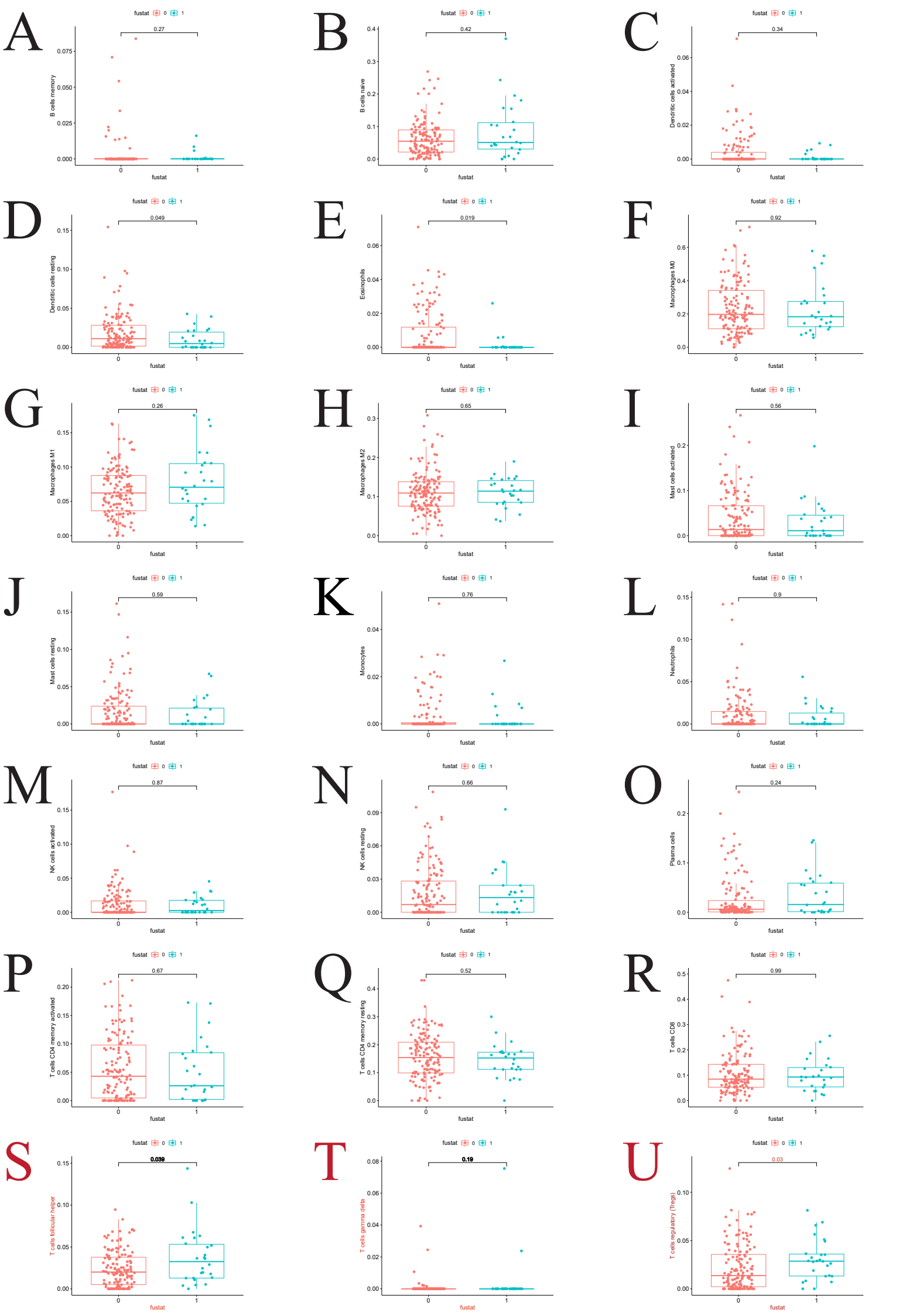

Supplement: Supplementary Materials — Figure s1: functional enrichment analysis results of differential autophagy genes in CRC. (A, B) Significantly enriched Gene Ontology terms of differential autophagy genes in CRC. BP: biological process; CC: cellular component; MF: molecular function. (C, D) Significantly enriched Kyoto Encyclopedia of Genes and Genomes pathways of differential autophagy genes in CRC. Figure s2: correlation between tumor-infiltrating immune cells where red represented the positive correlation while green represented the negative correlation in CRC. Figure s3: the correlation between the CRC immune microenvironment and fustat. Figure s4: the correlation between the CRC immune microenvironment and gender. Figure s5: the correlation between the CRC immune microenvironment and pathological stage M. Figure s6: the correlation between the CRC immune microenvironment and pathological stage N. Figure s7: the correlation between the CRC immune microenvironment and cancer stage. Figure s8: the correlation between the CRC immune microenvironment and pathological stage T. Figure s9: the correlation of DAPK1 with 21 types of TII cell distribution. Figure s10: the correlation of MAP1LC3C with 19 types of TII cell distribution. Figure s11: the correlation of PELP1 with 19 types of TII cell distribution. Figure s12: the correlation of risk score with 19 types of TII cell distribution. [file 2055676.f1.zip › 2055676.f3.pdf]

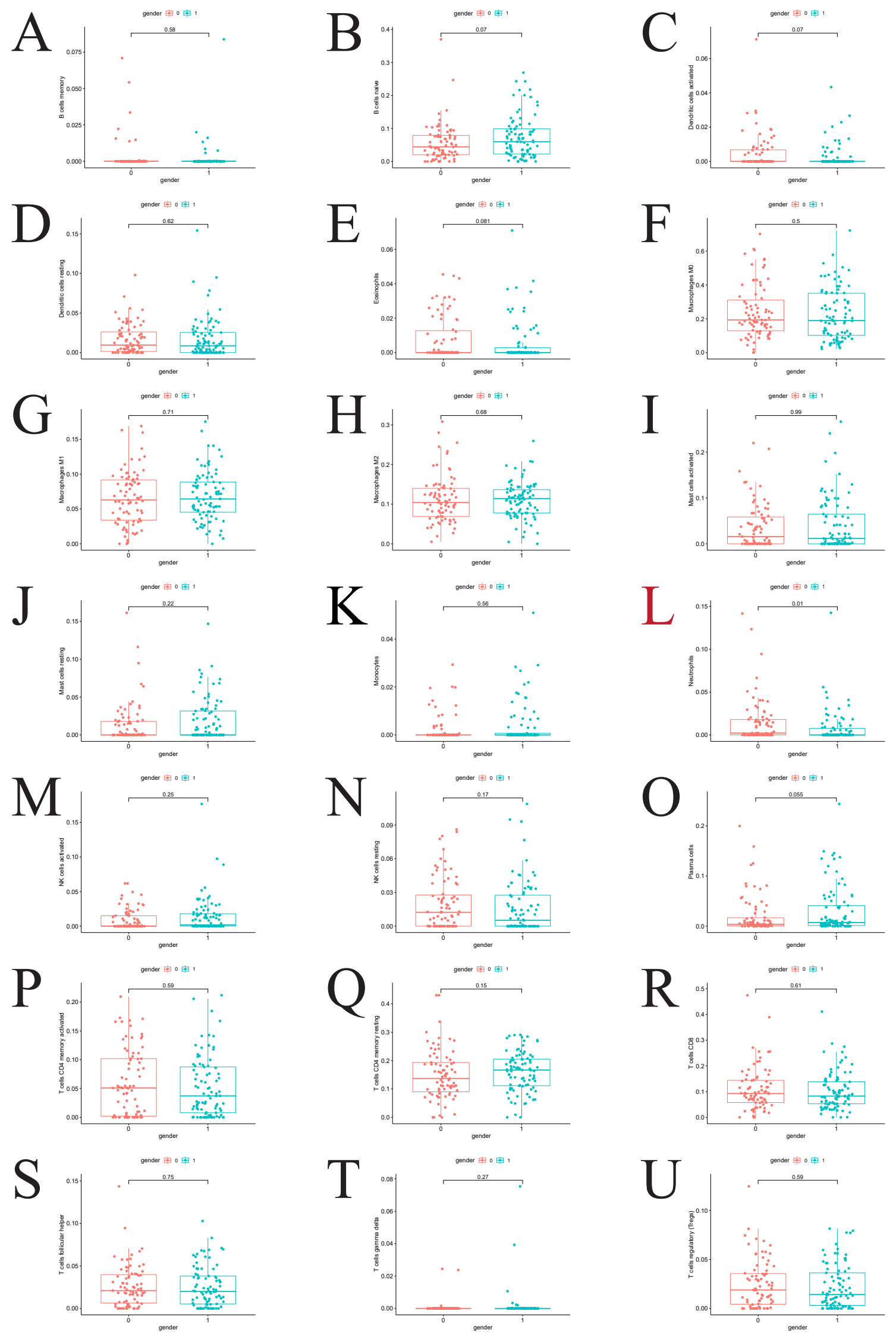

Supplement: Supplementary Materials — Figure s1: functional enrichment analysis results of differential autophagy genes in CRC. (A, B) Significantly enriched Gene Ontology terms of differential autophagy genes in CRC. BP: biological process; CC: cellular component; MF: molecular function. (C, D) Significantly enriched Kyoto Encyclopedia of Genes and Genomes pathways of differential autophagy genes in CRC. Figure s2: correlation between tumor-infiltrating immune cells where red represented the positive correlation while green represented the negative correlation in CRC. Figure s3: the correlation between the CRC immune microenvironment and fustat. Figure s4: the correlation between the CRC immune microenvironment and gender. Figure s5: the correlation between the CRC immune microenvironment and pathological stage M. Figure s6: the correlation between the CRC immune microenvironment and pathological stage N. Figure s7: the correlation between the CRC immune microenvironment and cancer stage. Figure s8: the correlation between the CRC immune microenvironment and pathological stage T. Figure s9: the correlation of DAPK1 with 21 types of TII cell distribution. Figure s10: the correlation of MAP1LC3C with 19 types of TII cell distribution. Figure s11: the correlation of PELP1 with 19 types of TII cell distribution. Figure s12: the correlation of risk score with 19 types of TII cell distribution. [file 2055676.f1.zip › 2055676.f4.pdf]

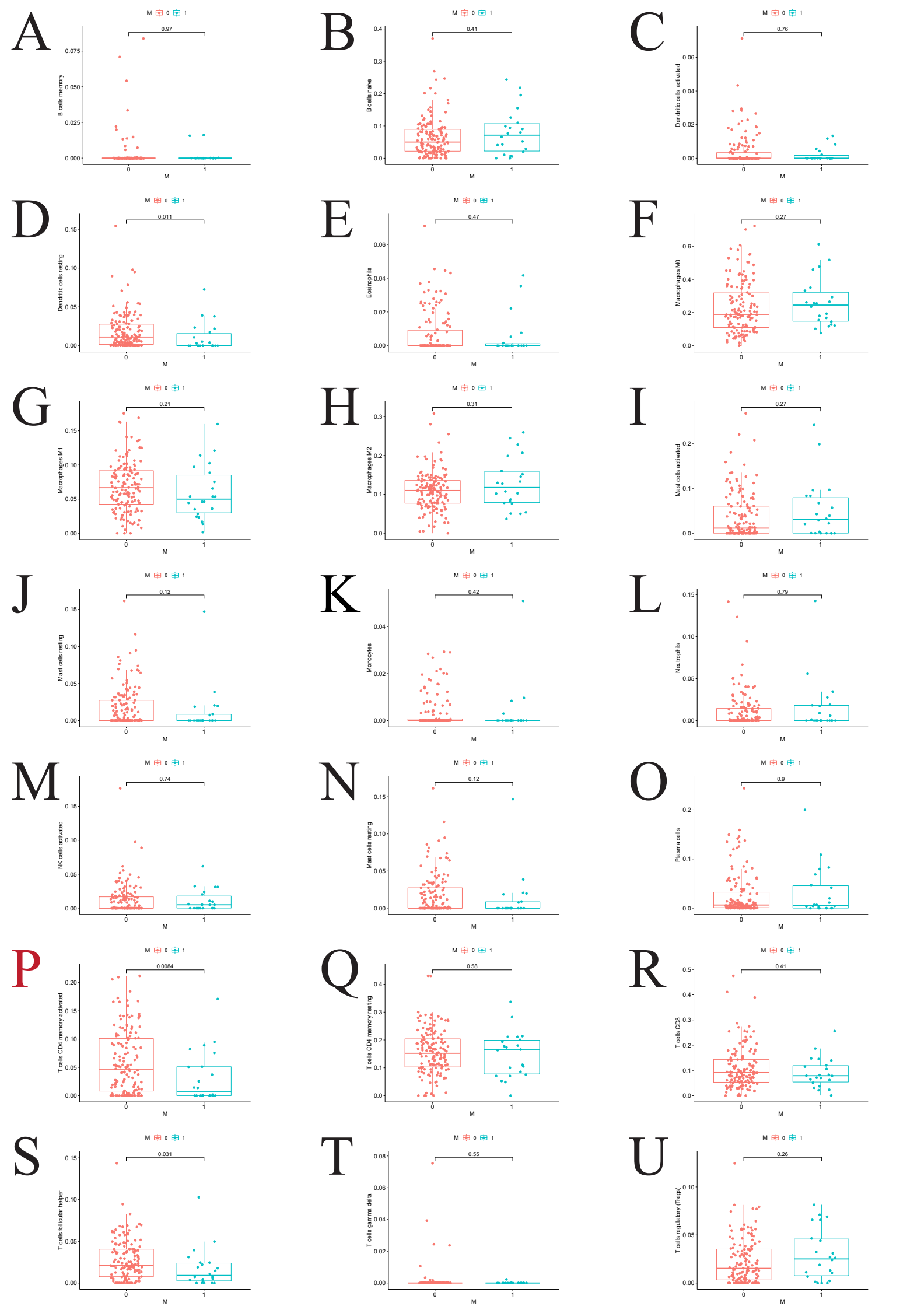

Supplement: Supplementary Materials — Figure s1: functional enrichment analysis results of differential autophagy genes in CRC. (A, B) Significantly enriched Gene Ontology terms of differential autophagy genes in CRC. BP: biological process; CC: cellular component; MF: molecular function. (C, D) Significantly enriched Kyoto Encyclopedia of Genes and Genomes pathways of differential autophagy genes in CRC. Figure s2: correlation between tumor-infiltrating immune cells where red represented the positive correlation while green represented the negative correlation in CRC. Figure s3: the correlation between the CRC immune microenvironment and fustat. Figure s4: the correlation between the CRC immune microenvironment and gender. Figure s5: the correlation between the CRC immune microenvironment and pathological stage M. Figure s6: the correlation between the CRC immune microenvironment and pathological stage N. Figure s7: the correlation between the CRC immune microenvironment and cancer stage. Figure s8: the correlation between the CRC immune microenvironment and pathological stage T. Figure s9: the correlation of DAPK1 with 21 types of TII cell distribution. Figure s10: the correlation of MAP1LC3C with 19 types of TII cell distribution. Figure s11: the correlation of PELP1 with 19 types of TII cell distribution. Figure s12: the correlation of risk score with 19 types of TII cell distribution. [file 2055676.f1.zip › 2055676.f5.pdf]

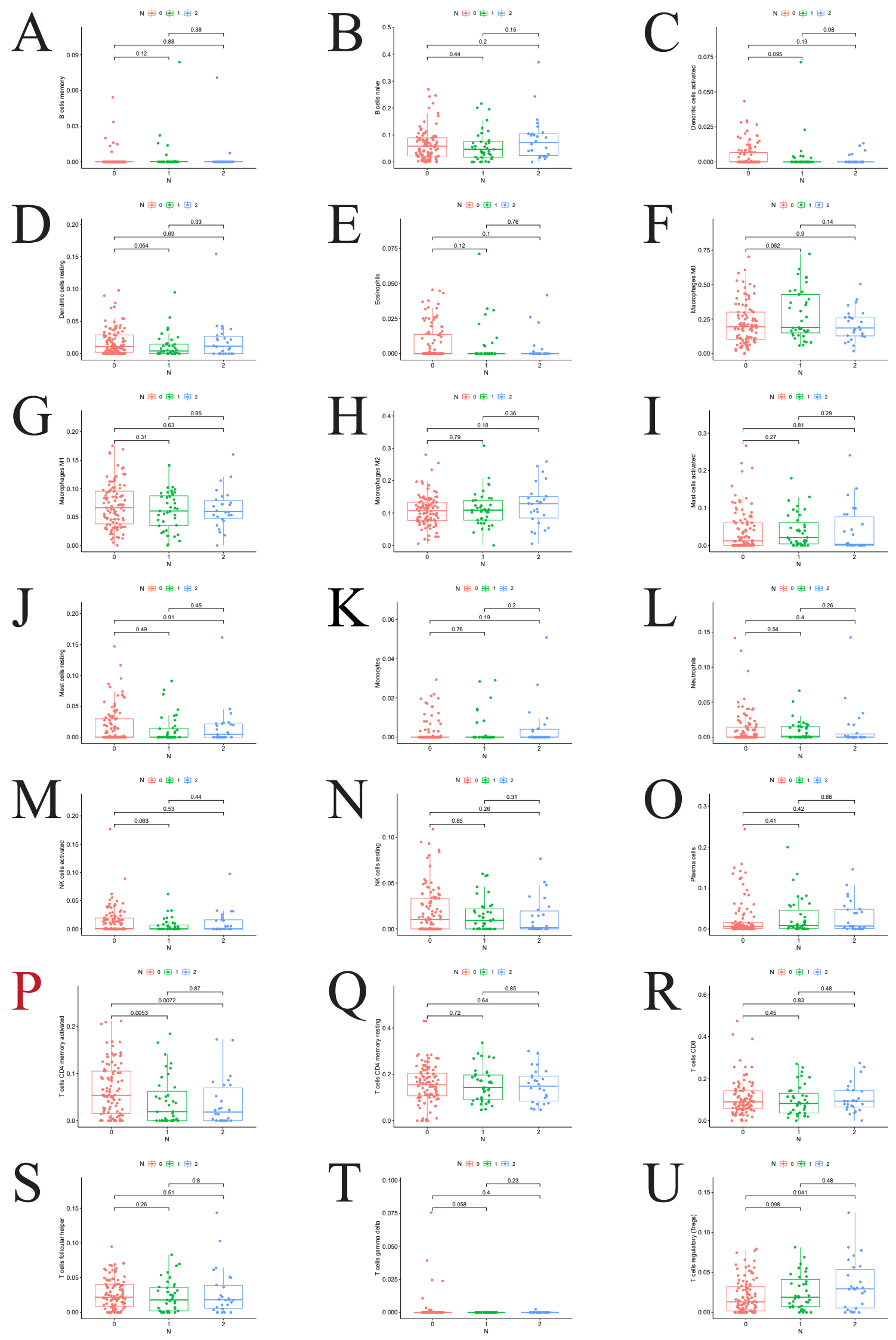

Supplement: Supplementary Materials — Figure s1: functional enrichment analysis results of differential autophagy genes in CRC. (A, B) Significantly enriched Gene Ontology terms of differential autophagy genes in CRC. BP: biological process; CC: cellular component; MF: molecular function. (C, D) Significantly enriched Kyoto Encyclopedia of Genes and Genomes pathways of differential autophagy genes in CRC. Figure s2: correlation between tumor-infiltrating immune cells where red represented the positive correlation while green represented the negative correlation in CRC. Figure s3: the correlation between the CRC immune microenvironment and fustat. Figure s4: the correlation between the CRC immune microenvironment and gender. Figure s5: the correlation between the CRC immune microenvironment and pathological stage M. Figure s6: the correlation between the CRC immune microenvironment and pathological stage N. Figure s7: the correlation between the CRC immune microenvironment and cancer stage. Figure s8: the correlation between the CRC immune microenvironment and pathological stage T. Figure s9: the correlation of DAPK1 with 21 types of TII cell distribution. Figure s10: the correlation of MAP1LC3C with 19 types of TII cell distribution. Figure s11: the correlation of PELP1 with 19 types of TII cell distribution. Figure s12: the correlation of risk score with 19 types of TII cell distribution. [file 2055676.f1.zip › 2055676.f6.pdf]

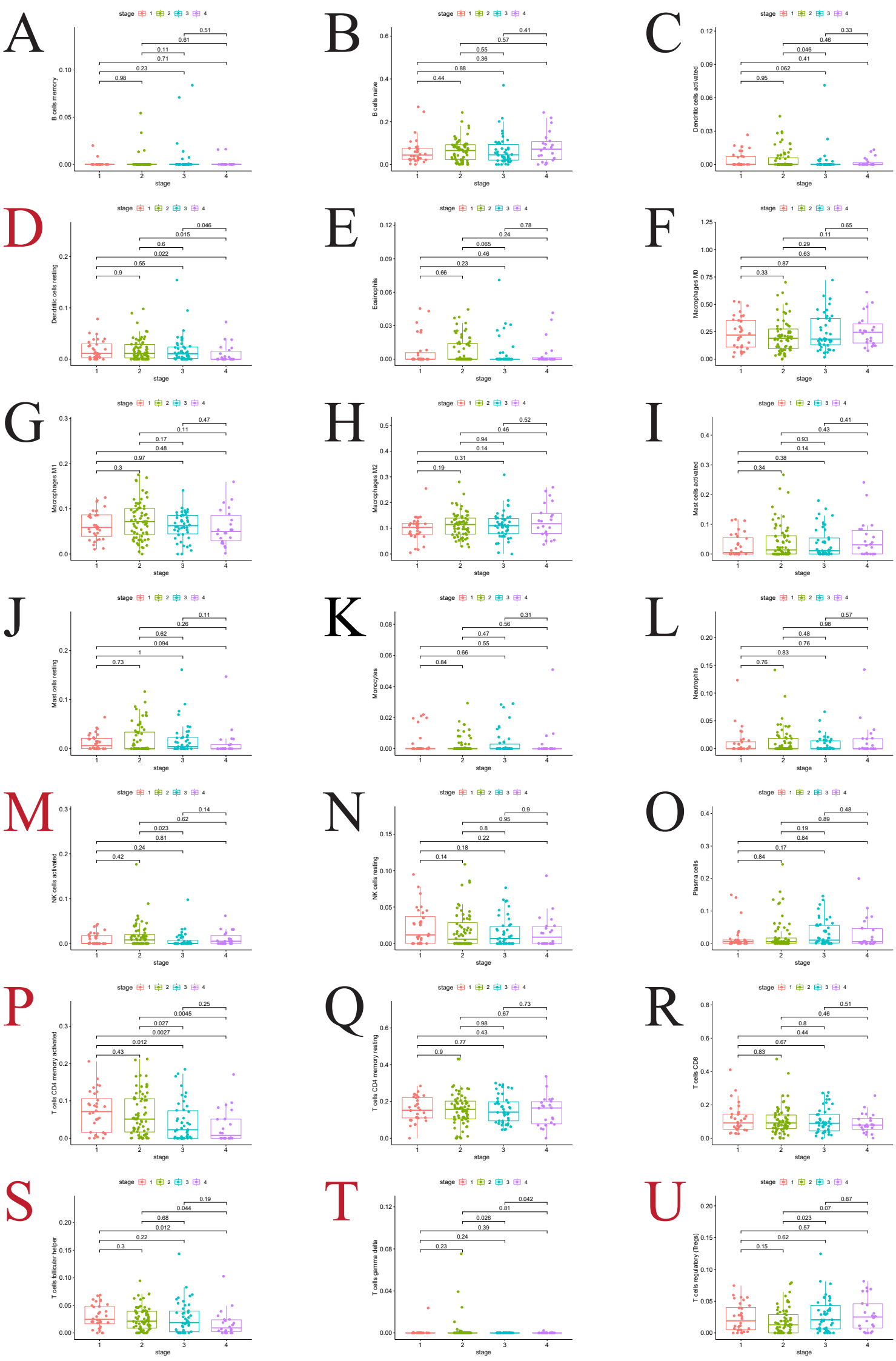

Supplement: Supplementary Materials — Figure s1: functional enrichment analysis results of differential autophagy genes in CRC. (A, B) Significantly enriched Gene Ontology terms of differential autophagy genes in CRC. BP: biological process; CC: cellular component; MF: molecular function. (C, D) Significantly enriched Kyoto Encyclopedia of Genes and Genomes pathways of differential autophagy genes in CRC. Figure s2: correlation between tumor-infiltrating immune cells where red represented the positive correlation while green represented the negative correlation in CRC. Figure s3: the correlation between the CRC immune microenvironment and fustat. Figure s4: the correlation between the CRC immune microenvironment and gender. Figure s5: the correlation between the CRC immune microenvironment and pathological stage M. Figure s6: the correlation between the CRC immune microenvironment and pathological stage N. Figure s7: the correlation between the CRC immune microenvironment and cancer stage. Figure s8: the correlation between the CRC immune microenvironment and pathological stage T. Figure s9: the correlation of DAPK1 with 21 types of TII cell distribution. Figure s10: the correlation of MAP1LC3C with 19 types of TII cell distribution. Figure s11: the correlation of PELP1 with 19 types of TII cell distribution. Figure s12: the correlation of risk score with 19 types of TII cell distribution. [file 2055676.f1.zip › 2055676.f7.pdf]

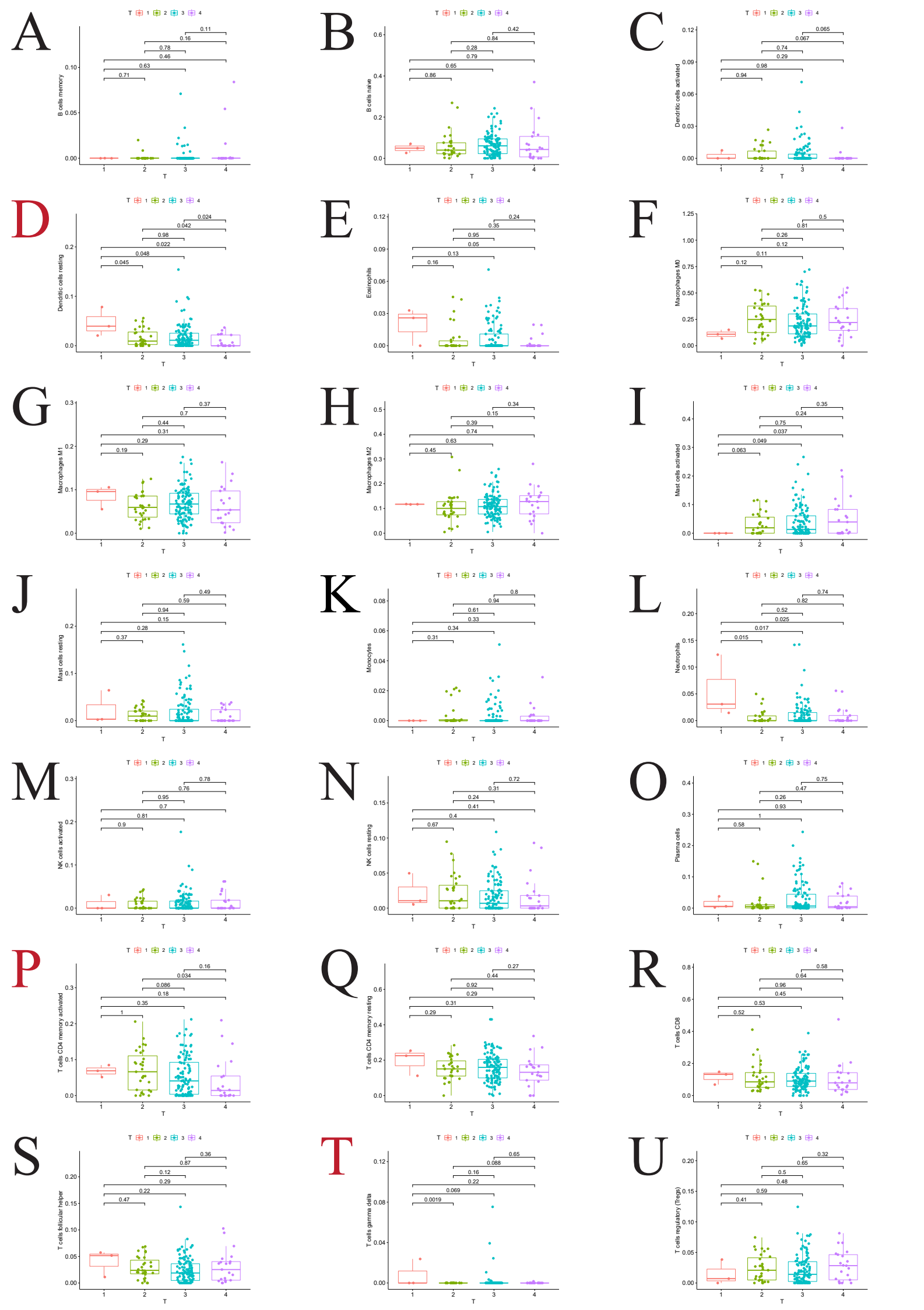

Supplement: Supplementary Materials — Figure s1: functional enrichment analysis results of differential autophagy genes in CRC. (A, B) Significantly enriched Gene Ontology terms of differential autophagy genes in CRC. BP: biological process; CC: cellular component; MF: molecular function. (C, D) Significantly enriched Kyoto Encyclopedia of Genes and Genomes pathways of differential autophagy genes in CRC. Figure s2: correlation between tumor-infiltrating immune cells where red represented the positive correlation while green represented the negative correlation in CRC. Figure s3: the correlation between the CRC immune microenvironment and fustat. Figure s4: the correlation between the CRC immune microenvironment and gender. Figure s5: the correlation between the CRC immune microenvironment and pathological stage M. Figure s6: the correlation between the CRC immune microenvironment and pathological stage N. Figure s7: the correlation between the CRC immune microenvironment and cancer stage. Figure s8: the correlation between the CRC immune microenvironment and pathological stage T. Figure s9: the correlation of DAPK1 with 21 types of TII cell distribution. Figure s10: the correlation of MAP1LC3C with 19 types of TII cell distribution. Figure s11: the correlation of PELP1 with 19 types of TII cell distribution. Figure s12: the correlation of risk score with 19 types of TII cell distribution. [file 2055676.f1.zip › 2055676.f8.pdf]

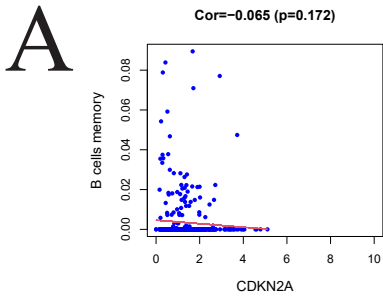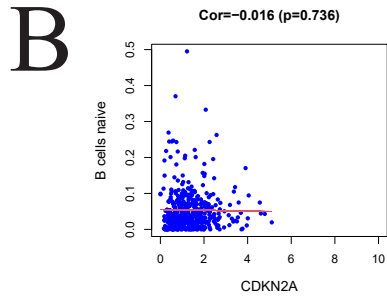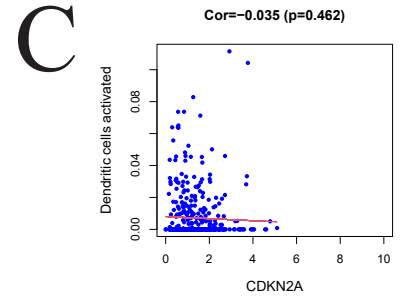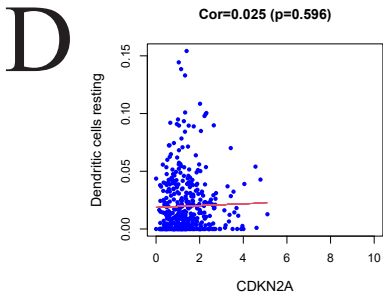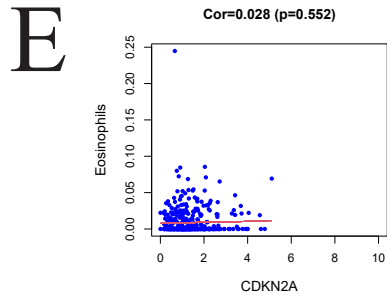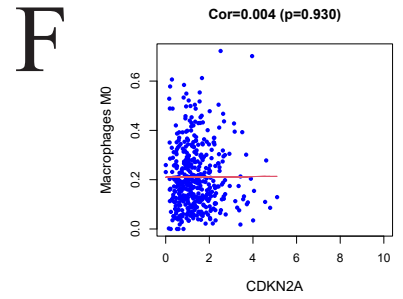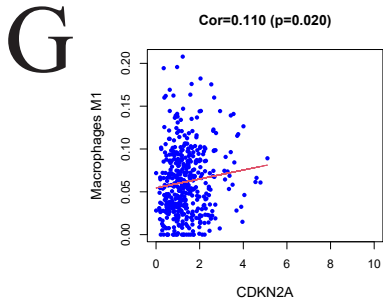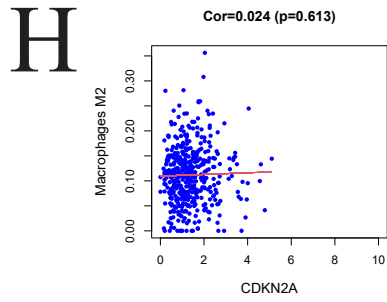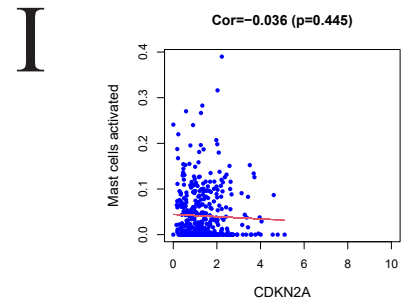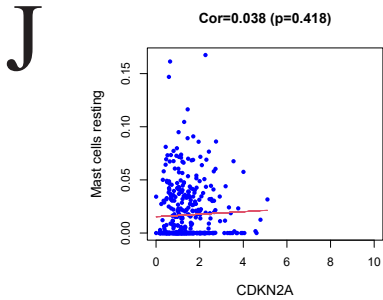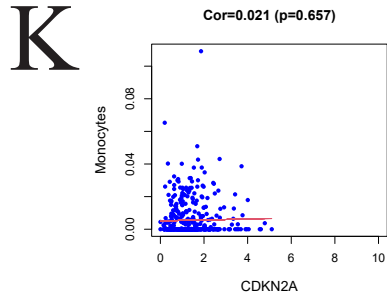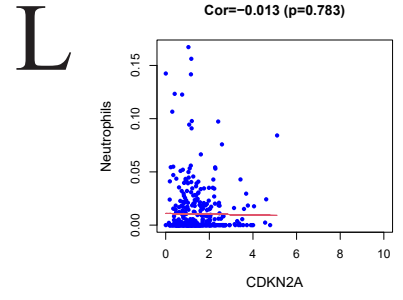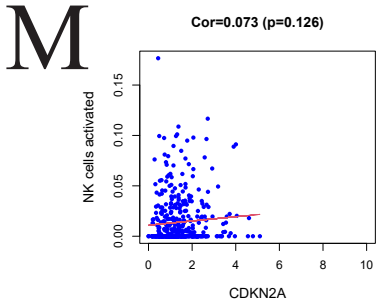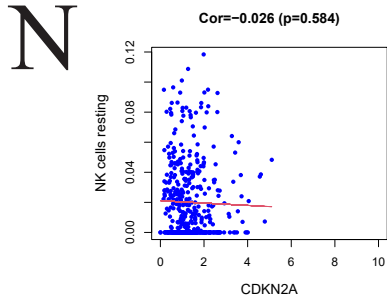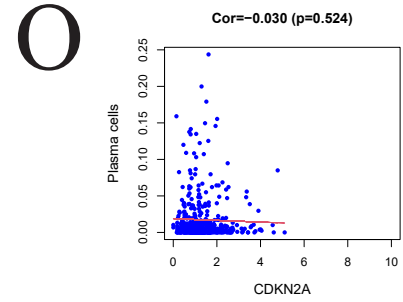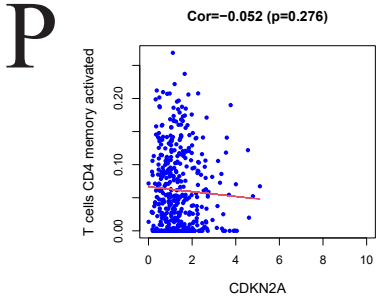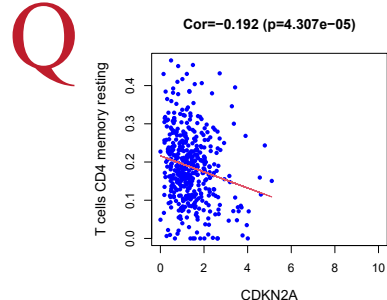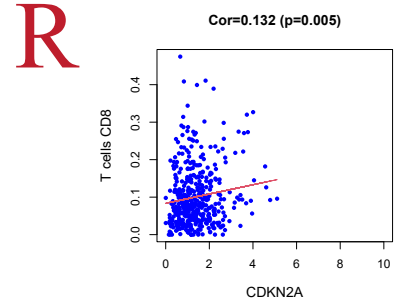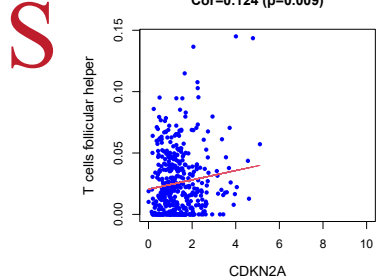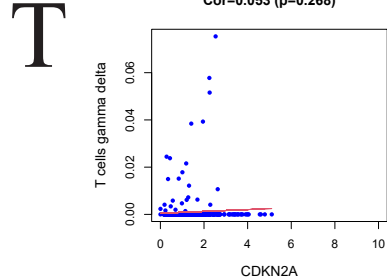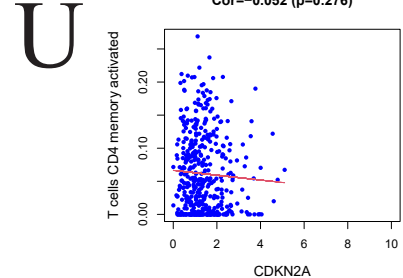

Supplement: Supplementary Materials — Figure s1: functional enrichment analysis results of differential autophagy genes in CRC. (A, B) Significantly enriched Gene Ontology terms of differential autophagy genes in CRC. BP: biological process; CC: cellular component; MF: molecular function. (C, D) Significantly enriched Kyoto Encyclopedia of Genes and Genomes pathways of differential autophagy genes in CRC. Figure s2: correlation between tumor-infiltrating immune cells where red represented the positive correlation while green represented the negative correlation in CRC. Figure s3: the correlation between the CRC immune microenvironment and fustat. Figure s4: the correlation between the CRC immune microenvironment and gender. Figure s5: the correlation between the CRC immune microenvironment and pathological stage M. Figure s6: the correlation between the CRC immune microenvironment and pathological stage N. Figure s7: the correlation between the CRC immune microenvironment and cancer stage. Figure s8: the correlation between the CRC immune microenvironment and pathological stage T. Figure s9: the correlation of DAPK1 with 21 types of TII cell distribution. Figure s10: the correlation of MAP1LC3C with 19 types of TII cell distribution. Figure s11: the correlation of PELP1 with 19 types of TII cell distribution. Figure s12: the correlation of risk score with 19 types of TII cell distribution. [file 2055676.f1.zip › 2055676.f9.pdf]
